# Supplementary material for: Photoredox-Catalyzed Reduction of Halogenated Arenes in Water by Amphiphilic Polymeric Nanoparticles
Source: Molecules. 2021 Sep 28;26(19):5882. doi: 10.3390/molecules26195882 (PMC8512223; doi:10.3390/molecules26195882)
Supplement: Supplementary file 1 [file molecules-26-05882-s001.zip › molecules-1388004-supplementary.pdf]

Supporting Information for:

Photoredox catalyzed reduction of halogenated arenes in water by amphiphilic polymeric nanoparticles

Fabian Eisenreich[1], Tom H. R. Kuster[1], David van Krimpen[1] and Anja R. A. Palmans[1,*]

[1] Laboratory of Macromolecular and Organic Chemistry and Institute for Complex Molecular Systems, Eindhoven University of Technology, P.O. Box 513, 5600 MB Eindhoven, The Netherlands.
* Correspondence: a.palmans@tue.nl

Table of Contents

1. Methods and equipment 4
NMR-Spectrometer 4
MALDI-TOF-MS 4
UV/VIS spectroscopy 4
DLS 4
GPC 4
Dry Solvent System 4
Dialysis 4
LEDs 4
Automated Column Chromatography 5
2. Polymer synthesis 6
Pentafluorophenyl acrylate (M1) 6
Poly(pentafluorophenyl acrylate) (P1) 6
End-cap modification poly(pentafluorophenyl acrylate) (P_{200}) 7
Poly(pentafluorophenyl acrylate) (P_{100}) 7
3. Acridinium synthesis 8
3,3’-Oxybis(*tert*-butylbenzene) (A1) 8
Methyl 2,4,6-trimethylbenzoate (A2) 8
3,6-Di-*tert*-butyl-9-mesitylxanthylium tetrafluoroborate (A3) 9
10-(4-(2-((*tert*-Butoxycarbonyl)amino)ethyl)phenyl)-3,6-di-*tert*-butyl-9-mesitylacridin-10-ium tetrafluoroborate (A4) 10
10-(4-(2-Aminoethyl)phenyl)-3,6-di-*tert*-butyl-9-mesitylacridin-10-ium tetrafluoroborate (ACR-NH_2) 11
2-(4-(10H-Phenothiazin-10-yl)phenyl)ethan-1-amine (PTH-NH2) 12
4. Polymer Functionalization 12
Functionalization of poly(pentafluorophenyl acrylate) to PN1 12
Functionalization of poly(pentafluorophenyl acrylate) to PN2 13
Functionalization of poly(pentafluorophenyl acrylate) to PN3 14
Functionalization of poly(pentafluorophenyl acrylate) to PN4 15
Functionalization of poly(pentafluorophenyl acrylate) to PN5 16
Functionalization of poly(pentafluorophenyl acrylate) to PN6 17
SEC traces of P1 and P_{200} 18
5. PN characterization 18
Intermediate functionalization conversion 18
SEC traces of PN1-PN6 22

Dynamic Light Scattering ... 23
UV/VIS spectra ... 24
6. Photoredox catalysis ... 25
Irradiation set up ... 25
Reaction mechanisms for the dehalogenation of aromatic halides ... 26
Procedure *para*-substituted aryl halide reaction ... 27
Conversion 4-chlorobenzonitrile (S1) ... 28
Conversion 4-chloroanisole (S2) ... 28
Conversion 4-chlorotoluene (S3) ... 29
Conversion 4-chlorobenzotrifluoride (S4) ... 29
Conversion methyl-4-chlorobenzoate (S5) ... 30
Procedure selectivity reaction ... 31
Conversion 1-chloro-4-iodobenzene (S6) ... 31
Conversion 1-bromo-4-chlorobenzene (S7) ... 32
Qualitative analysis 2-bromo-4-chloro-1-iodobenzene (S8) ... 33
Procedure for C-C cross coupling reactions ... 34
References ... 35
NMR spectra ... 36

1. Methods and equipment

NMR-Spectrometer

A Bruker 400 MHz Ultrashield spectrometer (^{1}H at 400 MHz, ^{13}C at 100 MHz and ^{19}F at 376 MHz) was used to measure the NMR spectra at 25 °C. The compound of interest was dissolved in $CDCl_3$, from Cambridge Isotope Laboratories, with the internal standard for ^{1}H and ^{13}C. (^{1}H: δ($CDCl_3$) = 7.26 ppm, δ($CDCl_3$) = 77.16 ppm). Multiplicities are indicated with the following abbreviations: singlet (s), doublet (d), doublet of doublet of doublets (ddd), triplet (t), quartet (q), multiplet (m) and broad (br). The obtained NMR spectra were processed using MestReNova v14.0.1-23559.

MALDI-TOF-MS

A Bruker Autoflex Speed MALDI-TOF was used to determine the Matrix assisted laser desorption/ionization time of light mass spectra (MALDI-TOF-MS) of the compound of interest. The MALDI-TOF uses either α-cyano-4-hydroxycinnamic acid (CHCA) or *trans*-2-[3-(4-*tert*-butylphenyl)-2-methyl-2-propenylidene]malononitrile (DCTB) as matrix.

UV/VIS spectroscopy

UV/VIS absorption spectra were performed on a JASCO V-650 spectrometer with a JASCO CTU-100 Circulating Thermoset Unit at 20 °C. The compound of interest was dissolved in acetonitrile (c = 0.04 $mmol_{catalyst}$/L).

DLS

Dynamic Light Scattering (DLS) spectra were obtained from a Malvern µV Zetasizer with a 830 nm laser and scatter angle of 90°. Disposable UV-transparent cuvettes from Sarstedt with a path length of 10 x 2 mm were used for the DLS measurements. Sample concentration are 1.0 mg/mL.

GPC

GPC measurements of poly(pentafluorophenyl) acrylate was performed on a Shimadzu Prominence-I LC-2030C 3D with a Shimadzu RID-20A refractive index detector. THF was used as the solvent, with a flow of 1.0 mL/min at an operating temperature of 40 °C. A mixed-C and mixed-D column in series (exclusion limit = 2000000 g/mol, 7.5 mm i.d. x 300 m), which has been calibrated using polystyrene (Polymer Laboratories), was used.

GPC measurements of functionalized polymers were performed on a PL-GPC-50 plus from Polymer Laboratories (Varian Inc. Company) with a refractive index detector. DMF with 10 mM LiBr was used as the solvent, with a flow of 1.0 mL/min and at an operating temperature of 50 °C. The implemented column was a Shodex GPC-KD-804 column (exclusion limit = 400000 Da, 0.8 cm i.d. x 300 mm), which has been calibrated with poly(ethylene oxide) (Polymer Laboratories).

Dry Solvent System

Dry solvents were obtained from MBraun solvent purification system (MB SPS-800).

Dialysis

To purify the functionalized polymers, standard RC Tubing from Spectra/Por® with a molecular weight cutoff (MWCO) of 6–8 kDa was used.

LEDs

Ten light-emitting diodes (LEDs) of Intelligent LED solution model ILH-XQ01-S380-SC211-WIR200 (385 nm, 3.85 W UV LEDs) were used to illuminate the samples during the photoredox experiments.

Automated Column Chromatography

A Biotage Isolera® One was used to carry out automated column chromatography using a 150 gram Biotage Silica cartridge.

2. Polymer synthesis

Pentafluorophenyl acrylate (M1)

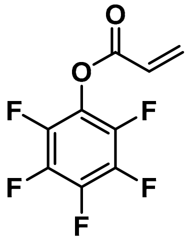

The monomer synthesis was carried out as described in literature.[1] A 250 mL round bottom flask with stirring bar was placed in an ice bath. Pentafluorophenol (16 g, 86.9 mmol, 1.0 eq.), triethylamine (14.5 mL, 104.3 mmol, 1.2 eq.) and 150 mL of dry diethyl ether were added to this flask. Subsequently, acryloyl chloride (8.4 mL, 104.3 mmol, 1.2 eq.) was added dropwise to the solution and the reaction mixture was left stirring in the ice bath for five minutes. Afterwards, the ice bath was removed and the reaction was stirred for 18 h at room temperature. The mixture was filtered through a paper filter. The filtrate was then purified via flash column chromatography (SiO_2, heptane) and the desired monomer **M1** (9.72 g, 40.82 mmol, 47% yield) was obtained as a colorless oil.

^{1}H NMR (400 MHz, $CDCl_3$, 25 °C): δ = 6.72 (d, *J* = 17.1 Hz, 1H, CH_{vinyl}), 6.38 (dd, *J* = 17.3, 10.5 Hz, 1H, CH_{vinyl}), 6.18 (d, *J* = 10.5 Hz, 1H, CH_{vinyl}) ppm.

^{13}C NMR (100 MHz, $CDCl_3$, 25 °C): δ = 161.6, 142.6, 140.8, 140.1, 139.1, 138.3, 136.6, 135.1, 125.2, 125.0 ppm.

^{19}F NMR (376 MHz, $CDCl_3$, 25 °C): δ = -153.4 (m), -159.01 (t, *J* = 21.3 Hz), -163.37 (m).

Poly(pentafluorophenyl acrylate) (P1)

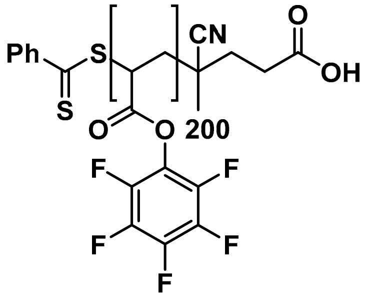

A dry Schlenk flask with stirring bar and septum cap was predried in a drying oven. Under inert conditions, pentafluorophenyl acrylate **M1** (4.0 g, 16.8 mmol, 1 equiv.), cyano-4-(phenyl-carbonothioylthio)pentanoic acid (16.4 mg, 58.8 µmol, 0.0035 equiv.) and azobisisobutyronitrile (0.965 mg, 5.88 µmol, 0.00035 equiv.) were dissolved in 3.5 mL of dry 1,4-dioxane and transferred into the Schlenk flask. After degassing the solution with argon for 45 minutes, the Schlenk flask was placed in the preheated oil bath at 80 °C. Monomer conversion was determined via ^{19}F NMR spectroscopy by taking a sample from the reaction mixture. At 5.5 h the conversion reached 70%, which corresponds to a degree of polymerization of approximately 200. The polymerization was halted by placing the Schlenk flask into liquid nitrogen. The polymer was precipitated three times from 500 mL of ice-cold pentane and filtered off using a glass filter. The residue on the filter was collected and dried in a vacuum oven at 50 °C for 18 h. Finally the desired poly(pentafluorophenyl acrylate) **P1** (1.87 g, $M_{w,theoretical}$ = 48 kg/mol, 39.1 µmol, 67% yield) was collected as a pink solid compound.

GPC (THF): M_n = 28003, $Đ$ = 1.17

^{1}**H NMR** (400 MHz, $CDCl_3$, 25 °C): δ = 3.09 (br), 2.50 (br), 1.97 (br) ppm.

^{19}**F NMR** (376 MHz, $CDCl_3$, 25 °C): δ = -153.3 (br, 2F, CF_{aryl}), -156.78 (br, 1F, CF_{aryl}), -162.19 (br, 2F, CF_{aryl}) ppm.

End-cap modification poly(pentafluorophenyl acrylate) (P_{200})

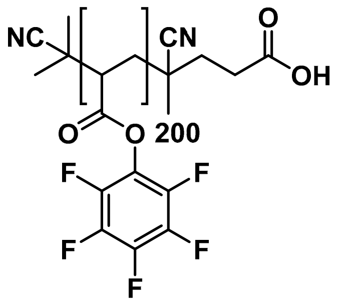

A dry Schlenk flask with stirring bar was predried in a drying oven. Under inert conditions, poly(pentafluorophenyl acrylate) **P1** (1.8 g, 38.0 µmol, 1.0 eq.), azobisisobutyronitrile (62.4 mg, 380 µmol, 10.0 eq.) and lauroyl peroxide (30.3 g, 76.0 µmol, 2.0 eq.) were dissolve in 2.5 mL of dry 1,4-dioxane and transferred to the Schlenk flask. The reaction mixture was degassed for 45 minutes with argon and heated to 80 °C. The reaction was left running until the reaction mixture's color changed from pink to transparent. After 3 h, the polymer was precipitated three times in 500 mL of ice-cold pentane and filtered off using a glass filter. The residue on the filter is collected and dried in a vacuum oven at 50 °C for 18 h. Finally, the desired end-cap modified poly(pentafluorophenyl acrylate) $\mathbf{P_{200}}$(1.55 g, $M_{w,theoretical}$ = 48 kg/mol, 32.4 µmol, 85% yield) was obtained as a white solid compound.

GPC (THF): M_n = 29330, $Đ$ = 1.15

^{1}**H NMR** (400 MHz, $CDCl_3$, 25 °C): δ = 3.09 (br), 2.51 (br) ppm.

^{19}**F NMR** (376 MHz, $CDCl_3$, 25 °C): δ = -153.3(br, 2F, CF_{aryl}), -156.8 (br, 1F, CF_{aryl}), -162.2 (br, 2F, CF_{aryl}) ppm.

Poly(pentafluorophenyl acrylate) (P_{100})

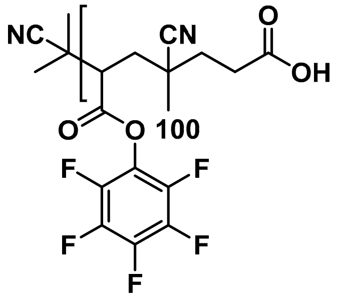

Poly(pentafluorophenyl acrylate) $\mathbf{P_{100}}$ with a degree of polymerization of 100 was prepared according to literature.[2]

3. Acridinium synthesis

3,3’-Oxybis(*tert*-butylbenzene) (A1)

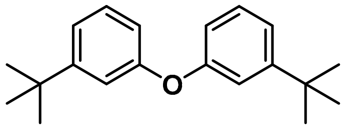

1-Bromo-3-(*tert*-butyl)benzene (2.5 g, 11.7 mmol, 1.0 eq.), 3-(*tert*-butyl)phenol (2.6 g, 17.6 mmol, 1.5 eq.), Cs_2CO_3 (7.6 g, 23.5 mmol, 2.0 eq.), CuI (223.4 mg, 1.2 mmol, 0.1 eq.), and 2,2,6,6-tetramethylheptane-3,5-dione (244.8 µL, 1.2 mmol, 0.1 eq.) were dissolved in 2.25 mL of DMF and the mixture was heated to 110 °C for 24 h. Afterwards, the reaction was cooled to room temperature and DMF was removed under reduced pressure. The reaction mixture was transferred to a 500 mL round bottom flask using 200 mL of Et_2O. Subsequently, the solution was filtered through celite and the organic phase was washed water (2x 100 mL) and brine (1x100 mL). Subsequently, the organic phase was dried over $MgSO_4$ and the crude product was purified using column chromatography (SiO_2, hexane/ethyl acetate). 3,3’-oxybis(tert-butylbenzene) **A1** (1.09 g, 3.86 mmol, 33% yield) was obtained as a colorless oil.

^{1}**H NMR** (400 MHz, $CDCl_3$, 25 °C): δ = 7.25 (t, *J* = 8.0 Hz, 2H), 7.12 (ddd, *J* = 8.0, 1.8, 1.1 Hz, 2H), 7.09 (t, *J* = 2.2 Hz, 2H), 6.79 (ddd, *J* = 8.0, 2.5, 1.0 Hz, 2H), 1.30 (s, 18H) ppm.

Methyl 2,4,6-trimethylbenzoate (A2)

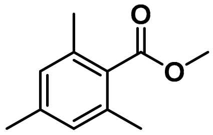

2,4,6-Trimethylbenzoic acid (6.57 mg, 40 mmol, 1.0 eq.) and K_2CO_3 (8.29 mg, 60 mmol, 1.5 eq.) were dissolved in 50 mL of DMF. Methyl iodide (3.0 mL, 48 mmol, 1.2 eq.) was added dropwise to the reaction mixture and stirred for 24 h at room temperature. Then, 300 mL of water was added to the reaction mixture and aqueous phase was extracted Et_2O (3x300 mL). The combined organic phases were washed water (3x150 mL) and brine (1x150 mL) and dried over $MgSO_4$. The solution was passed through silica using a 1:1 ethyl acetate/pentane mixture as eluent and the solvent was removed under educed pressure. Methyl 2,4,6-trimethylbenzoate **A2** (5.71 g, 32 mmol, 80% yield) was obtained as a pale yellow oil.

^{1}**H NMR** (400 MHz, $CDCl_3$, 25 °C) δ = 6.84 (s, 2H), 3.88 (s, 3H), 2.27 (br, 9H).

3,6-Di-*tert*-butyl-9-mesitylxanthylium tetrafluoroborate (A3)

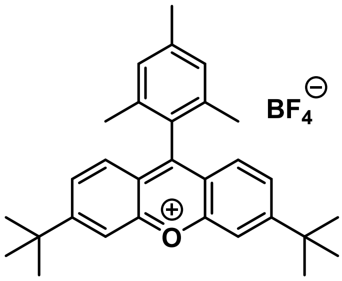

The synthesis was conducted according to literature[3]. 3,3'-Oxybis(*tert*-butylbenzene) **A1** (1.0 g, 3.9 mmol, 1 eq.), TMEDA (1.2 mL, 7.9 mmol, 2.05 eq.) and 3.82 mL of anhydrous *n*-hexane were added to the oven dried flask under argon. The solution was cooled in an ice bath for 20 minutes. 1.4 M sec-butyllithium in cyclohexane (5.7 mL, 7.9 mmol, 2.05 eq.) was added dropwise. Subsequently, the ice bath was removed and the reaction was left to run for 4 h. Methyl 2,4,6-trimethylbenzoate **A2** (695.7 mg, 3.9 mmol, 1.01 eq.) was added at -78 °C and the reaction was stirred for 16 h at room temperature. Afterwards, the reaction was quenched with 25 mL of water and stirred rigorously for 30 minutes. The mixture was diluted with 100 mL of Et_2O and the organic layer was separated from the water layer. The organic phase was washed with water (2x150 mL) and brine (1x150 mL). The organic layer was transferred to a 250 mL round bottom flask with stirring bar. Concentrated HCl solution (1.6 mL) was added dropwise whilst the solution was being stirred vigorously for 45 minutes, creating a bright yellow precipitate. Subsequently, the mixture was diluted with 150 mL of water and the layers were separated. The organic phase was washed with 150 mL of water until water layer became colorless. The combined aqueous layers were treated with $NaBF_4$ (1.3 g, 11.6 mmol, 3.0 eq.), forming a bright yellow precipitate. This aqueous phase was extracted with 150 mL of DCM, until the organic layer was colorless. The organic layers were combined in an Erlenmeyer and HBF_4 ● Et_2O-complex (0.471 mL, 3.9 mmol, 1.0 eq.) was added. The solution was mixed until homogeneity and subsequently washed with water (1x 150 mL) and 1M aq. $NaBF_4$ (1x100 mL). The organic layer was dried with $NaBF_4$ and filtered off the solution with a glass filter. Residual DCM was removed under reduced pressure. The remaining compound was triturated 3x with pentane and the product was dried for 18 h in vacuo at room temperature. 3,6-di-*tert*-butyl-9-mesitylxanthylium tetrafluoroborate **A3** (1.09 g, 2.18 mmol, 56% yield) was obtained as a yellow-red solid.

^{1}H NMR (400 MHz, $CDCl_3$, 25 °C): δ = 8.48 (s, 2H), 7.90 (d, *J* = 8.8 Hz, 2H), 7.74 (d, *J* = 9.0 Hz, 2H), 7.16 (s, 2H), 2.47 (s, 3H), 1.85 (s, 6H), 1.55 (s, 18H) ppm.

^{13}C NMR (100 MHz, $CDCl_3$, 25 °C): δ = 174.3, 171.0, 158.6, 141.3, 135.4, 129.1, 128.5, 127.6, 122.1, 116.9, 37.6, 30.5, 21.3, 20.2 ppm.

^{19}F NMR (376 MHz, $CDCl_3$, 25 °C): δ = -154.1 (m), -154.2 (m) ppm.

MALDI-TOF m/z calculated for $C_{30}H_{35}O$ $[M-BF_4]^+$ 411.27, found 411.37.

10-(4-(2-((*tert*-Butoxycarbonyl)amino)ethyl)phenyl)-3,6-di-*tert*-butyl-9-mesitylacridin-10-ium tetrafluoroborate (A4)

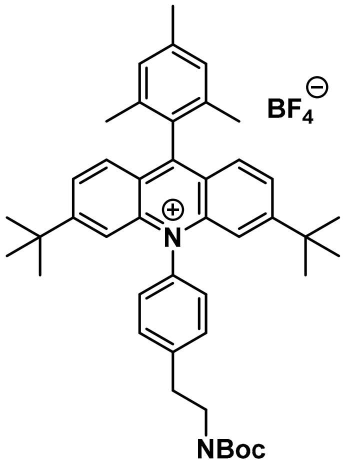

The synthesis was adapted from literature[3]. 3,6-Di-*tert*-butyl-9-mesitylxanthylium tetrafluoroborate **A3** (900 mg, 1.8 mmol, 1.0 eq.) was added to an oven dried flask and dry DCM (3.6 mL) was added. Subsequently, acetic acid (309.8 µL, 5.4 mmol, 3.0 eq.) and NEt_3 (377.5 µL, 2.7 mmol, 1.5 eq.) were added to the solution. *tert*-Butyl (4-aminophenethyl)carbamate (853.4 mg, 2.6 mmol, 2 eq.) was dissolved separately in 2 mL of dry DCM and the solution was added dropwise to the reaction mixture. Next, the flask was wrapped in aluminum foil and the reaction mixture was stirred for 16 h at room temperature. The reaction mixture was subsequently washed with water (1x150 mL) and brine (1x50 mL). HBF_4•Et_2O-complex (220 µL, 1.8 mmol, 1 eq.) was added to the organic phase and swirled until homogeneity. The organic phase was washed with water (1x 100 mL) and 1M aq. $NaBF_4$ (1x100 mL) and afterwards dried over $NaBF_4$. After removing the solvent, the solid compound was triturated 3x with 1:2 Et_2O:pentane and the obtained product was dried for 18 h in vacuo at room temperature. 10-(4-(2-((*tert*-Butoxycarbonyl)-amino)ethyl)phenyl)-3,6-di-*tert*-butyl-9-mesitylacridin-10-ium tetrafluoroborate **A4** (1.1436 g, 1.60 mmol, 88% yield) was obtained as a dark yellow solid.

^{1}H NMR (400 MHz, $CDCl_3$, 25 °C): δ = 7.83 (t, *J* = 8.0 Hz, 2H), 7.79 (m, 4H), 7.61 (d, *J* = 8.0 Hz, 2H), 7.44 (s, 2H), 7.17 (s, 2H), 5.03 (br, 1H), 3.57 (q, *J* = 8.0 Hz, 2H), 3.14 (t, *J* = 7.4 Hz, 2H), 2.49 (s, 3H), 1.85 (s, 6H), 1.30 (s, 18H) ppm.

^{13}C NMR (100 MHz, $CDCl_3$, 25 °C): δ = 163.8, 162.3, 156.2, 143.9, 142.2, 140.3, 136.0, 134.8, 132.0, 129.2, 129.0, 128.3, 127.8, 127.5, 124.0, 115.1, 41.7, 36.8, 36.7, 36.1, 30.2, 28.5, 21.3, 20.2 ppm.

^{19}F NMR (376 MHz, $CDCl_3$, 25 °C): δ = -153.7 (m), -153.8 (m) ppm.

MALDI-TOF m/z calculated for $C_{43}H_{53}N_2O_2$ $[M-BF_4]^+$ 629.41, found 629.43.

10-(4-(2-Aminoethyl)phenyl)-3,6-di-*tert*-butyl-9-mesitylacridin-10-ium tetrafluoroborate (ACR-NH_2)

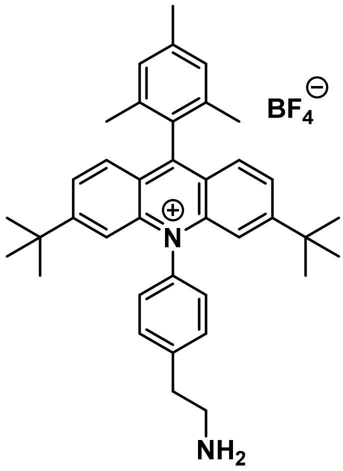

10-(4-(2-((*tert*-Butoxycarbonyl)amino)ethyl)phenyl)-3,6-di-tert-butyl-9-mesitylacridin-10-ium tetrafluoroborate **A4** (250.0 mg, 348.8 µmol, 1 eq.) was dissolved in 4M HCl in dioxane (5 mL, 17.4 mmol, 50 eq.) and the solution was stirred at room temperature. After 6 h total conversion was confirmed by TLC (heptane:ethanol 4:1) and 5.5 mL of 4M NaOH solution was added to the solution. The reaction mixture was stirred rigorously for 24 h at room temperature. Afterwards, the aqueous layer was extracted with DCM (4x25 mL) and the combined organic layers were washed with water (3x25 mL). 1M $NaBF_4$ solution (100 mL) was added to the organic layer and stirred vigorously for 45 minutes. The organic layer was separated from the aqueous phase and dried over $NaBF_4$. After removing the solvent under reduced pressure, the solid compound was triturated 3x with 1:2 Et_2O:pentane and dried for 18 h in vacuo at room temperature. 10-(4-(2-Aminoethyl)phenyl)-3,6-di-*tert*-butyl-9-mesitylacridin-10-ium tetrafluoroborate **ACR-NH_2** (193.46 mg, 313.76 µmol, 90% yield) was obtained as bright yellow solid.

^{1}H NMR (400 MHz, $CDCl_3$, 25 °C): δ = 8.01 (d, *J* = 8.1 Hz, 2H), 7.78 (d, *J* = 1.9 Hz, 4H), 7.63 – 7.54 (m, 2H), 7.47 (s, 2H), 7.16 (s, 2H), 6.88 (s, 3H), 3.72 (d, *J* = 10.5 Hz, 2H), 3.58 – 3.49 (m, 2H), 2.48 (s, 3H), 1.83 (s, 5H), 1.31 (s, 18H) ppm.

^{13}C NMR (100 MHz, $CDCl_3$, 25 °C): δ = 163.3, 161.3, 141.1, 140.5, 139.3, 134.9, 134.2, 131.7, 128.2, 128.0, 127.2, 126.9, 126.7, 122.9, 114.2, 40.6, 35.8, 31.9, 29.2, 21.3, 20.3 ppm.

^{19}F NMR (376 MHz, $CDCl_3$, 25 °C): δ = -150.8 (d, J = 19.6 Hz) ppm.

MALDI-TOF m/z calculated for C: m/z calculated for $C_{38}H_{45}N_2^+$ $[M-BF_4]^+$ 529.36, found 529.37.

2-(4-(10H-Phenothiazin-10-yl)phenyl)ethan-1-amine (PTH-NH2)

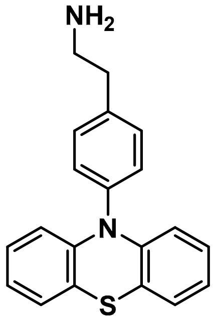

2-(4-(10H-phenothiazin-10-yl)phenyl)ethan-1-amine **PTH-NH₂** was prepared as described in literature[2].

4. Polymer Functionalization

Functionalization of poly(pentafluorophenyl acrylate) to PN1

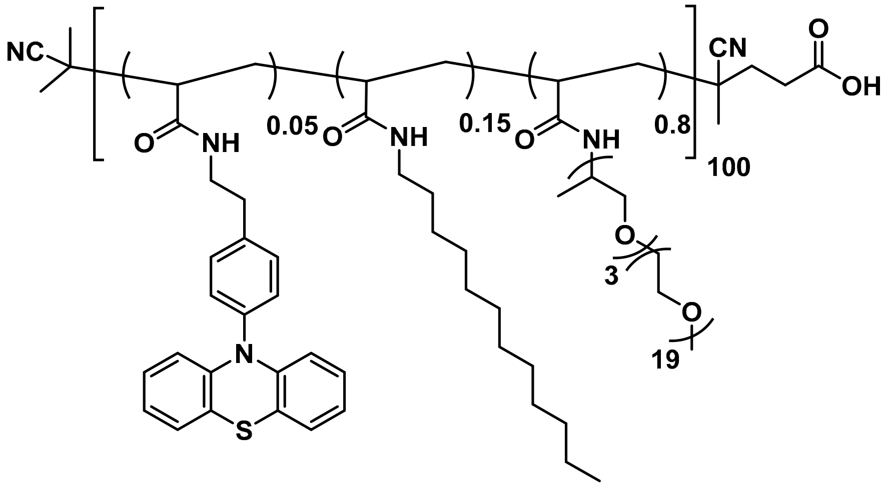

Under inert conditions, the endcap modified poly(pentafluorophenyl) acrylate 100mer **P3** (250 mg, 10.4 µmol, 1.0 eq.) was dissolved in 10 mL of dry THF. First, phenothiazine **A6** (16.6 mg, 52.1 µmol, 5.0 eq.) was added, then dodecylamine (29.0 mg, 156.2 µmol, 15.0 eq.), and finally an excess of Jeffamine® (1.63 g, 1.56 mmol, 150.0 eq.). After each addition step, the reaction mixture was left to stir for 18 h at 50 °C and the conversion of the functionalization was determined via ^{19}F NMR spectroscopy. After the entire polymer was functionalized, dialysis in THF (2x1 L) and methanol (1x1 L) was performed. Subsequently, the reaction mixture in the dialysis membrane was pipetted into a vail and dried in a vacuum oven for 18 h at 50 °C. Product **PN1** (842 mg, $M_{w,theoretical}$ = 90.0 kg/mol, 9.3 µmol, 89% yield) was obtained as a slightly yellow waxy solid.

GPC (DMF): M_n = 26.5, $Đ$ = 1.15

^{1}H NMR: see **Figure S32**.

Functionalization of poly(pentafluorophenyl acrylate) to PN2

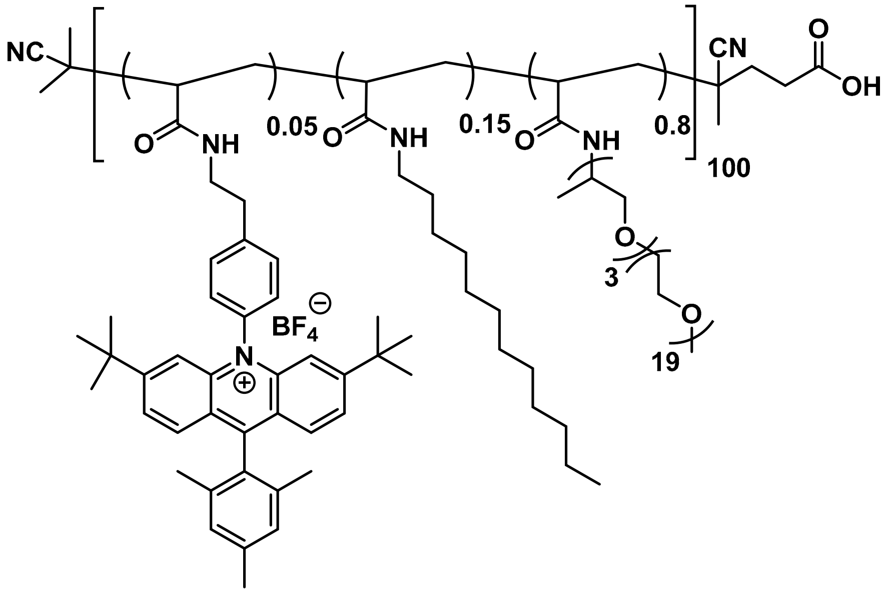

Under inert conditions, the endcap modified poly(pentafluorophenyl acrylate) 100mer $\mathbf{P_{100}}$ (100 mg, 1.0 eq.) was dissolved in 4 mL of dry THF. First, acridinium **A5** (12.95 mg, 5.0 eq.) and 50 µL of dry TMEDA were added. Then, dodecylamine (11.68 mg, 15 eq.) and finally an excess of Jeffamine® (666 mg, 150 eq.). After each addition step, the reaction mixture was left to stir for 18 h at 50 °C and the conversion of the functionalization was determined via ^{19}F NMR spectroscopy. After the entire polymer was functionalized, dialysis in THF (2x1 L) and methanol (1x1 L) was performed. Subsequently, the reaction mixture in the dialysis membrane was pipetted into a vail and dried in a vacuum oven for 18 h at 50 °C. Product **PN2** (340 mg, $M_{w,theoretical}$ = 91.5 kg/mol, 3.7 µmol, 89% yield) was obtained as a slightly yellow waxy solid.

GPC (DMF): M_n = 27.5, $Đ$ = 1.15

^{1}H NMR: see **Figure S33**.

Functionalization of poly(pentafluorophenyl acrylate) to PN3

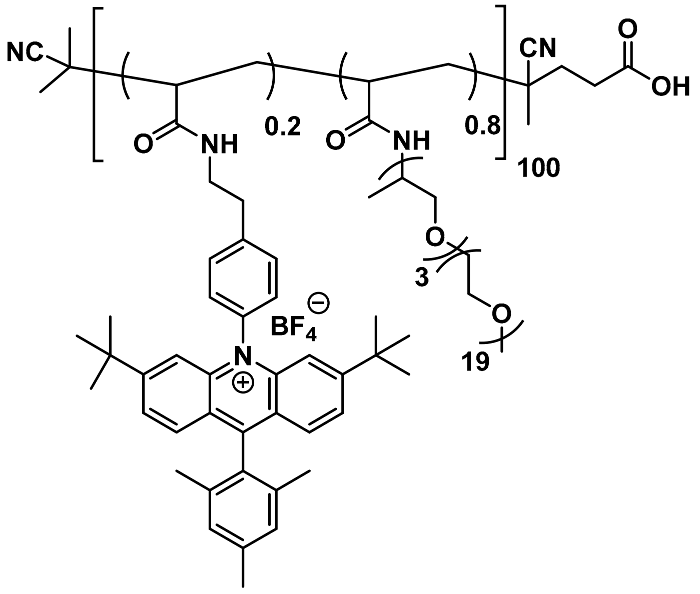

Under inert conditions, the endcap modified poly(pentafluorophenyl acrylate) 100mer **P3** (100 mg, 4.2 µmol, 1.0 eq.) was dissolved in 4 mL of dry THF. First, acridinium **A5** (51.4 mg, 83.3 µmol, 20 eq.) and 12.5 µL of dry TMEDA were added, followed by excess of Jeffamine® (651.3 mg, 624.9 µmol, 150 eq.). After each addition step, the reaction mixture was left to stir for 18 h at 50 °C and the conversion of the functionalization was determined via ^{19}F NMR spectroscopy. After the entire polymer was functionalized, dialysis in THF (2x1 L) and methanol (1x1 L) was performed. Subsequently, the reaction mixture in the dialysis membrane was pipetted into a vail and dried in a vacuum oven for 18 h at 50 °C. Product **PN3** (333 mg, $M_{w,theoretical}$ = 97.9 kg/mol, 3.4 µmol, 81% yield) was obtained as a yellow waxy solid.

GPC (DMF): M_n = 24062, $Đ$ = 1.19

^{1}H NMR: see **Figure S34**.

Functionalization of poly(pentafluorophenyl acrylate) to PN4

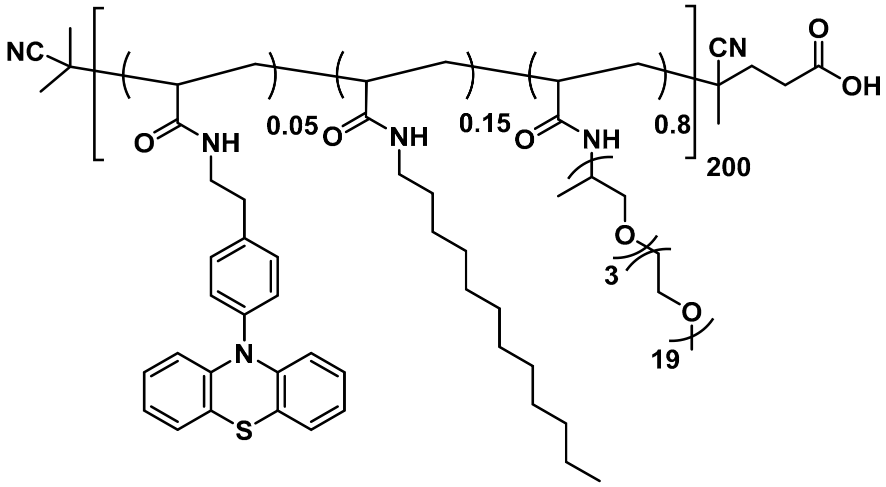

Under inert conditions, the endcap modified poly(pentafluorophenyl acrylate) 200mer **P2** (100 mg, 2.1 µmol, 1 eq.) was dissolved in 4 mL of dry THF. First, phenothiazine **A6** (6.7 mg, 20.9 µmol, 10 eq.) was added, then dodecylamine (11.6 mg, 62.7 µmol, 30 eq.) and finally an excess of Jeffamine® (653.9 mg, 627.4 µmol, 300 eq.). After each addition step, the reaction mixture was left to stir for 18 h at 50 °C and the conversion of the functionalization was determined via ^{19}F NMR spectroscopy. After the entire polymer was functionalized, dialysis in THF (2x1 L) and methanol (1x1 L) was performed. Subsequently, the reaction mixture in the dialysis membrane was pipetted into a vail and dried in a vacuum oven for 18 h at 50 °C. Product **PN4** (290.0 mg, $M_{w,theoretical}$ = 179.7 kg/mol, 1.6 µmol, 76% yield) was obtained as a slightly yellow waxy solid.

GPC (DMF): M_n = 42812, $Đ$ = 1.17

^{1}H NMR: see **Figure S35**.

Functionalization of poly(pentafluorophenyl acrylate) to PN5

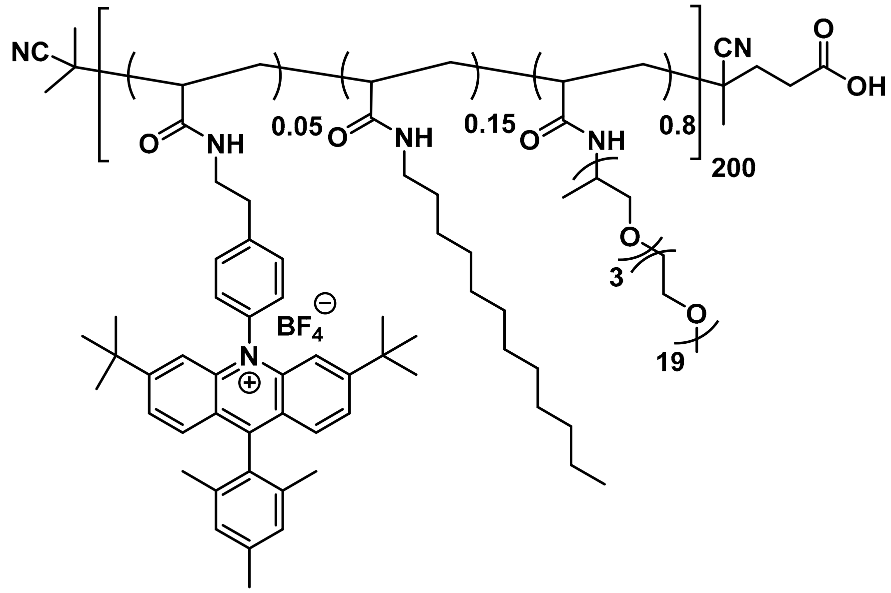

Under inert conditions, the endcap modified poly(pentafluorophenyl acrylate) 200mer **P2** (100 mg, 2.1 µmol, 1 eq.) was dissolved in 4 mL of dry THF. First, acridinium **A6** (12.9 mg, 20.9 µmol, 10 eq.) together with 3.1 µL of dry TMEDA was added. Then, dodecylamine (11.6 mg, 62.7 µmol, 30 eq.) and finally an excess of Jeffamine® (653.9 mg, 627.4 µmol, 300 eq.). After each addition step, the reaction mixture was left to stir for 18 h at 50 °C and the conversion of the functionalization was determined via ^{19}F NMR spectroscopy. After the entire polymer was functionalized, dialysis in THF (2x1 L) and methanol (1x1 L) was performed. Subsequently, the reaction mixture in the dialysis membrane was pipetted into a vail and dried in a vacuum oven for 18 h at 50 °C. Product **PN5** (309 mg, $M_{w,theoretical}$ = 182.7 kg/mol, 1.69 µmol, 80% yield) was obtained as a slightly yellow waxy solid.

GPC (DMF): M_n = 41226, $Đ$ = 1.24

^{1}H NMR: see **Figure S36**.

Functionalization of poly(pentafluorophenyl acrylate) to PN6

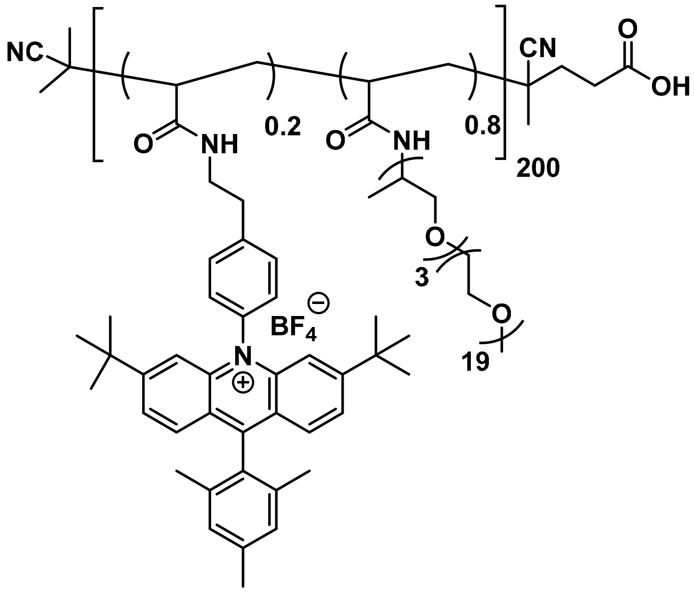

Under inert conditions, the endcap modified poly(pentafluorophenyl acrylate) 200mer **P2** (100 mg, 2.1 µmol, 1 eq.) was dissolved in 4 mL of dry THF. First, acridinium **A6** (51.6 mg, 83.7 µmol, 40 eq.) together with 12.5 µL of dry TMEDA was added, followed by an excess of Jeffamine® (653.9 mg, 627.4 µmol, 300 eq.). After each addition step, the reaction mixture was left to stir for 18 h at 50 °C and the conversion of the functionalization was determined via ^{19}F NMR spectroscopy. After the entire polymer was functionalized, dialysis in THF (2x1 L) and methanol (1x1 L) was performed. Subsequently, the reaction mixture in the dialysis membrane was pipetted into a vail and dried in a vacuum oven for 18 h at 50 °C. Product **PN6** (337 mg, $M_{w,theoretical}$ = 195.6 kg/mol, 1.72 µmol, 82% yield) was obtained as a yellow waxy solid.

GPC (DMF): M_n = 33705, $Đ$ = 1.31

^{1}H NMR: see **Figure S37**.

SEC traces of P1 and P_{200}

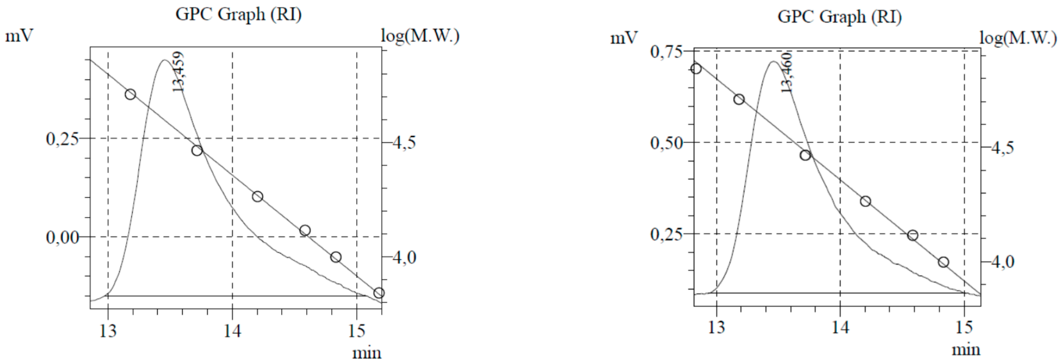

Total Average(RI)
Chromatogram Detector 2 Ch1

#	Mn	Mw	Mw/Mn
1	28003	32819	1,17200
	28003	32819	1,17200

Total Average(RI)
Chromatogram Detector 2 Ch1

#	Mn	Mw	Mw/Mn
1	29330	33693	1,14875
	29330	33693	1,14875

Figure S1: SEC traces of **P1** (left) and **P_{200}** (right) in THF calibrated with polystyrene standards.

5. PN characterization

Several techniques were used to determine different characteristics of the **PNs**. These results are summarized in this section.

Intermediate functionalization conversion

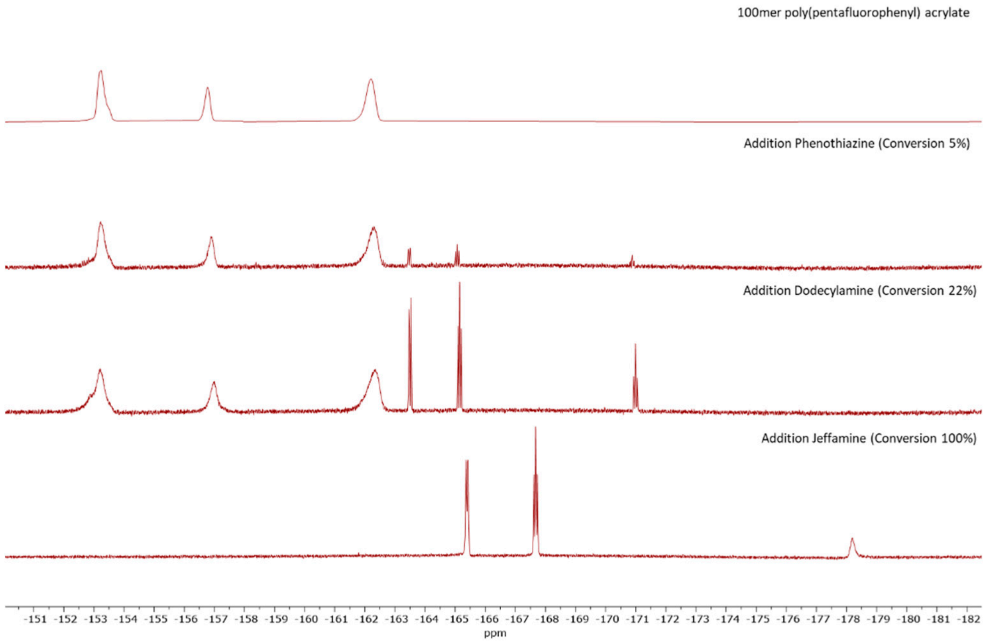

Figure S2: ^{19}F NMR spectra (376 MHz, $CDCl_3$, 25 °C) of each functionalization step of **PN1**. The conversion was determined by the ratio of pentafluorophenol which is connected to the polymer backbone (broad signals) and the free pentafluorophenol (sharp signals).

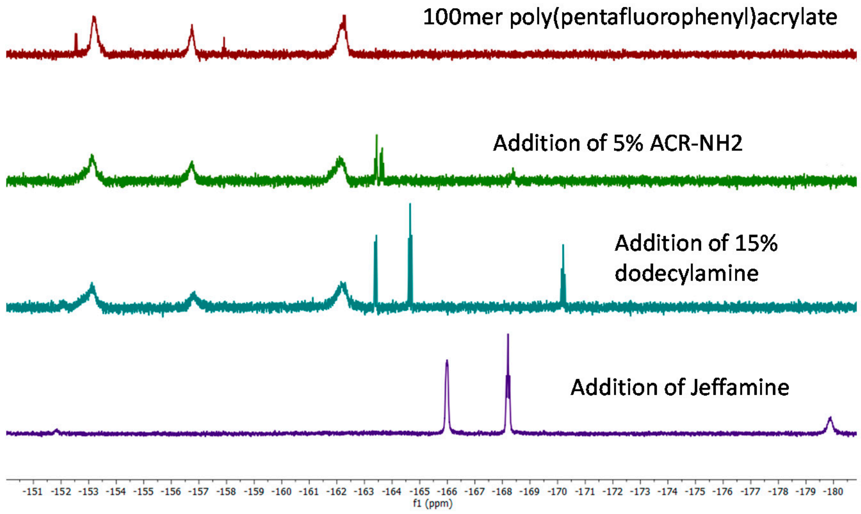

Figure S3: ^{19}F NMR spectra (376 MHz, $CDCl_3$, 25 °C) of each functionalization step of **PN2**. The conversion was determined by the ratio of pentafluorophenol which is connected to the polymer backbone (broad signals) and the free pentafluorophenol (sharp signals).

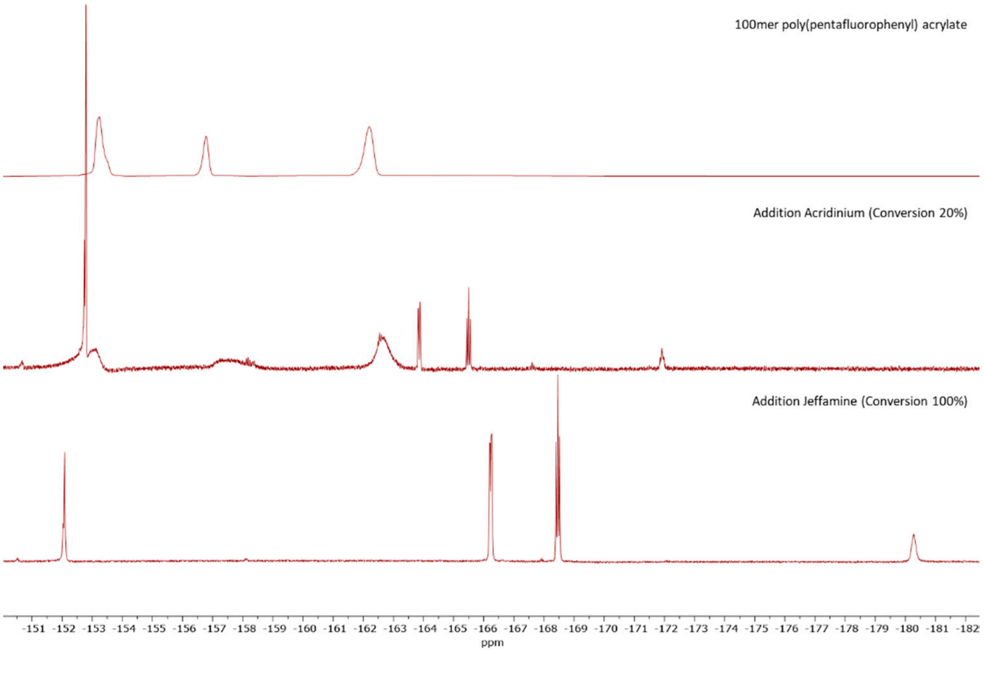

Figure S4: ^{19}F NMR spectra (376 MHz, $CDCl_3$, 25 °C) of each functionalization step of **PN3**. The conversion was determined by the ratio of pentafluorophenol which is connected to the polymer backbone (broad signals) and the free pentafluorophenol (sharp signals).

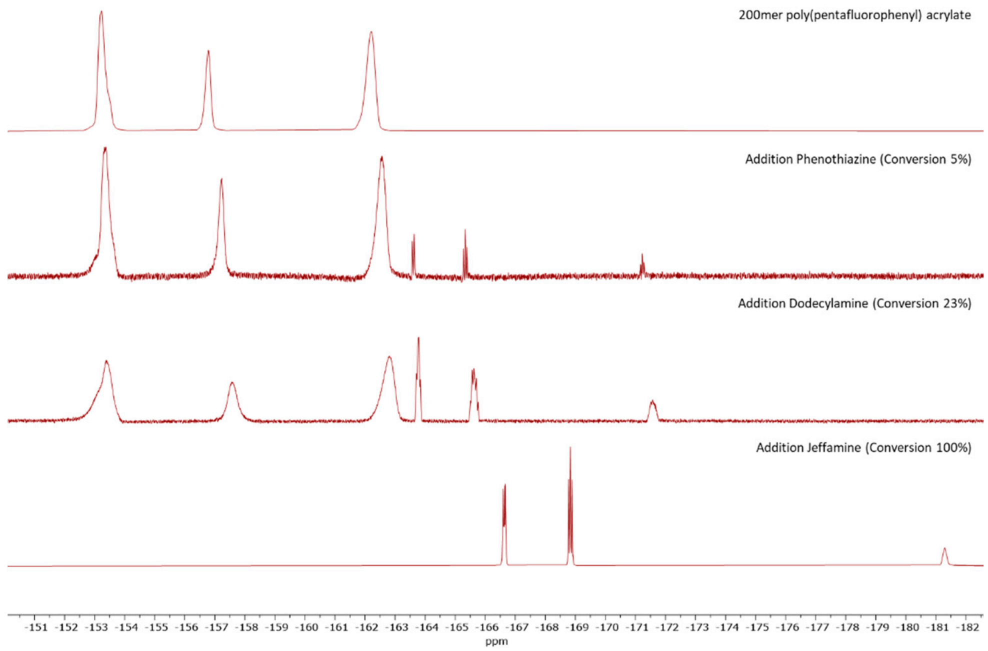

Figure S5: ^{19}F NMR spectra (376 MHz, $CDCl_3$, 25 °C) of each functionalization step of **PN4**. The conversion was determined by the ratio of pentafluorophenol which is connected to the polymer backbone (broad signals) and the free pentafluorophenol (sharp signals).

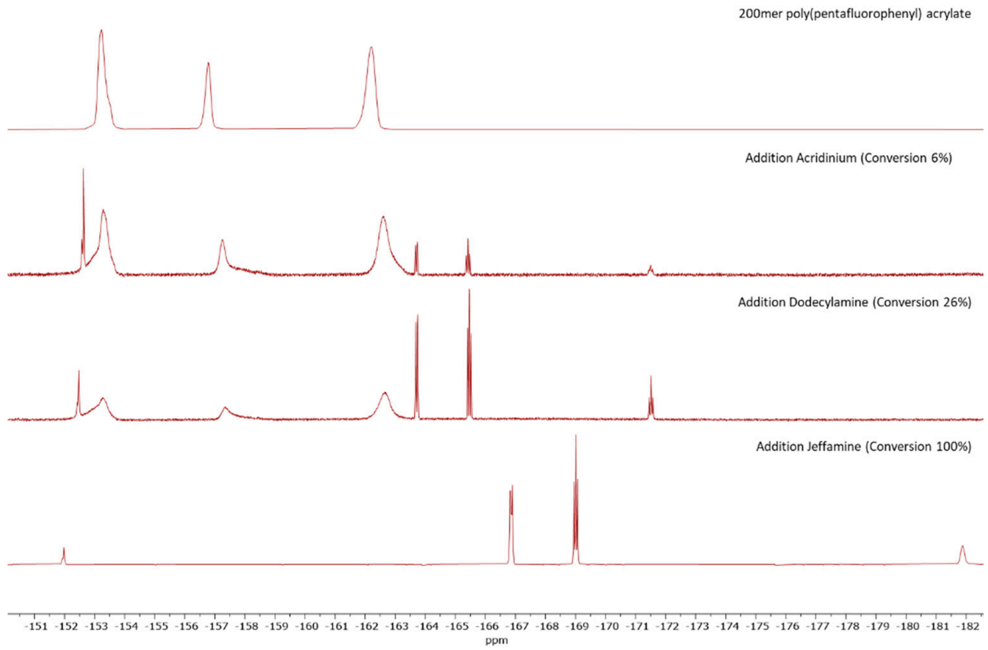

Figure S6: ^{19}F NMR spectra (376 MHz, $CDCl_3$, 25 °C) of each functionalization step of **PN5**. The conversion was determined by the ratio of pentafluorophenol which is connected to the polymer backbone (broad signals) and the free pentafluorophenol (sharp signals).

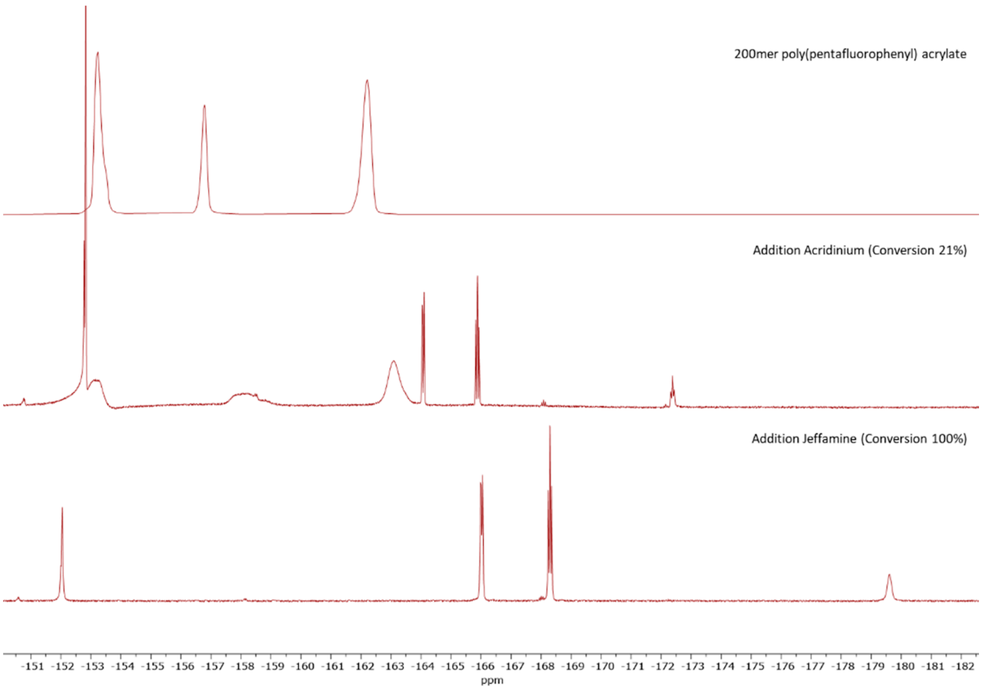

Figure S7: ^{19}F NMR spectra (376 MHz, $CDCl_3$, 25 °C) of each functionalization step of **PN6**. The conversion was determined by the ratio of pentafluorophenol which is connected to the polymer backbone (broad signals) and the free pentafluorophenol (sharp signals).

SEC traces of PN1-PN6

a)

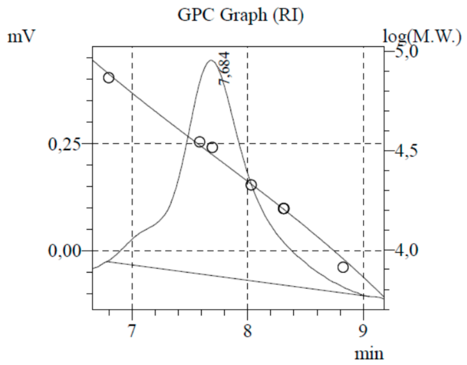

Total Average(RI)

Chromatogram Detector 2 Ch1

#	Mn	Mw	Mw/Mn
1	26545	30463	1,14760
	26545	30463	1,14760

d)

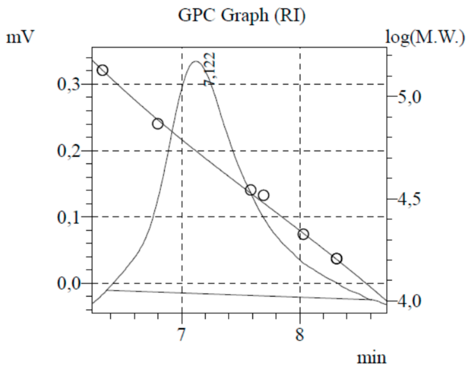

Total Average(RI)

Chromatogram Detector 2 Ch1

#	Mn	Mw	Mw/Mn
1	42812	50289	1,17465
	42812	50289	1,17465

b)

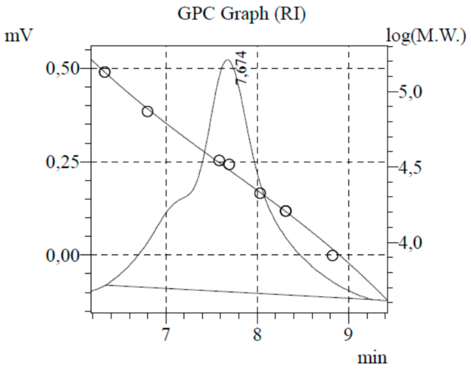

Total Average(RI)

Chromatogram Detector 2 Ch1

#	Mn	Mw	Mw/Mn
1	27518	35575	1,29278
	27518	35575	1,29278

e)

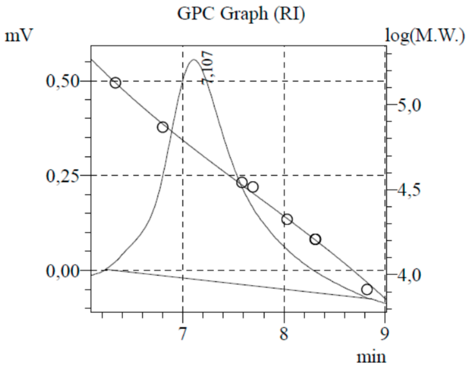

Total Average(RI)

Chromatogram Detector 2 Ch1

#	Mn	Mw	Mw/Mn
1	41226	50971	1,23638
	41226	50971	1,23638

c)

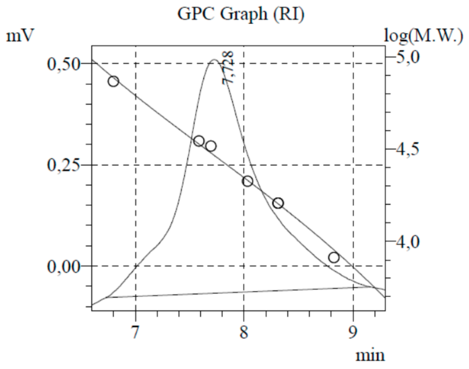

Total Average(RI)

Chromatogram Detector 2 Ch1

#	Mn	Mw	Mw/Mn
1	24062	28745	1,19460
	24062	28745	1,19460

f)

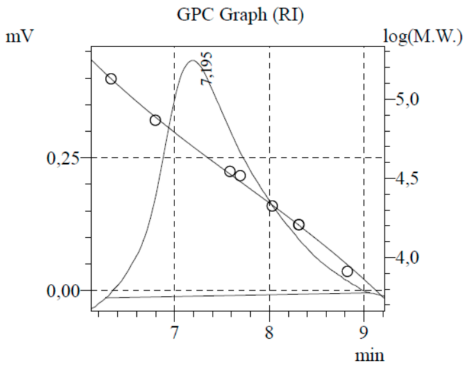

Total Average(RI)

Chromatogram Detector 2 Ch1

#	Mn	Mw	Mw/Mn
1	33705	44268	1,31341
	33705	44268	1,31341

Figure S8: SEC traces of **PN1**-**PN6** (a-f) in DMF with LiBr calibrated with poly(ethyleneoxide).

Dynamic Light Scattering

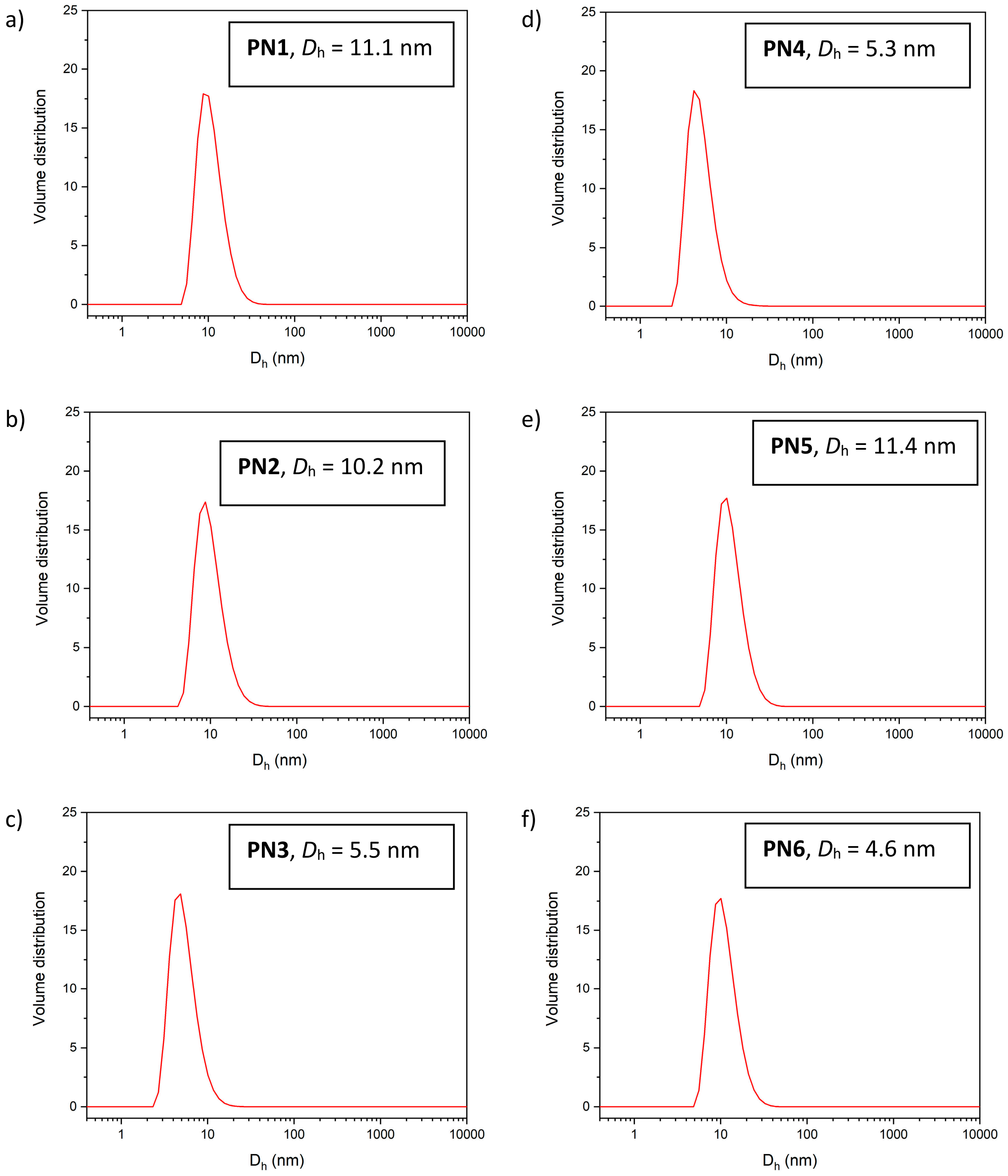

Figure S9: Dynamic light scattering (DLS) measurements of polymer solution ($c_{polymer}$ = 1.0 mg/ml) in water at 20 °C. a) DP = 100, 5% PTH **PN1** with an average D_h of 11.1 nm, b) DP = 100, 5% ACR **PN2** with an average D_h of 10.2 nm, c) DP = 100, 20% ACR **PN3** with an average D_h of 5.5 nm, d) DP = 200, 5% PTH **PN4** with an average D_h of 5.3 nm, e) **PN5** DP = 200, 5% ACR with an average D_h of 11.4 nm, f) DP = 200, 20% ACR **PN6** with an average D_h of 4.6 nm.

UV/VIS spectra

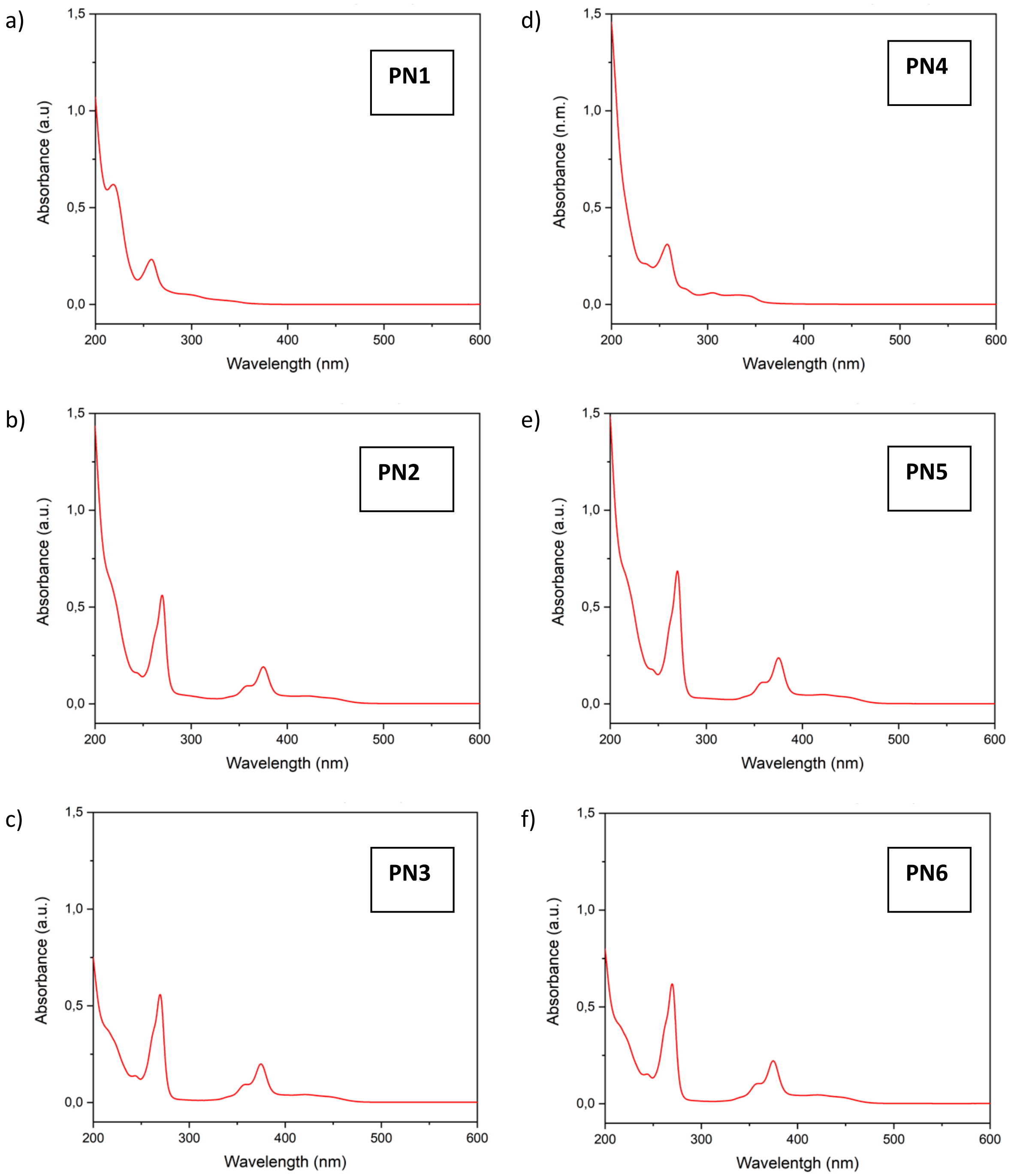

Figure S10: UV/VIS spectra of polymer solution in water ($c_{polymer}$ = 1.0 mg/mL) at 20 °C. The absorption profile of the polymer bound phenothiazine (a and d) and acridinium (b, c, e and f) align with free phenothiazine and acridinium absorption profiles reported in literature.[3,4]

6. Photoredox catalysis

Irradiation set up

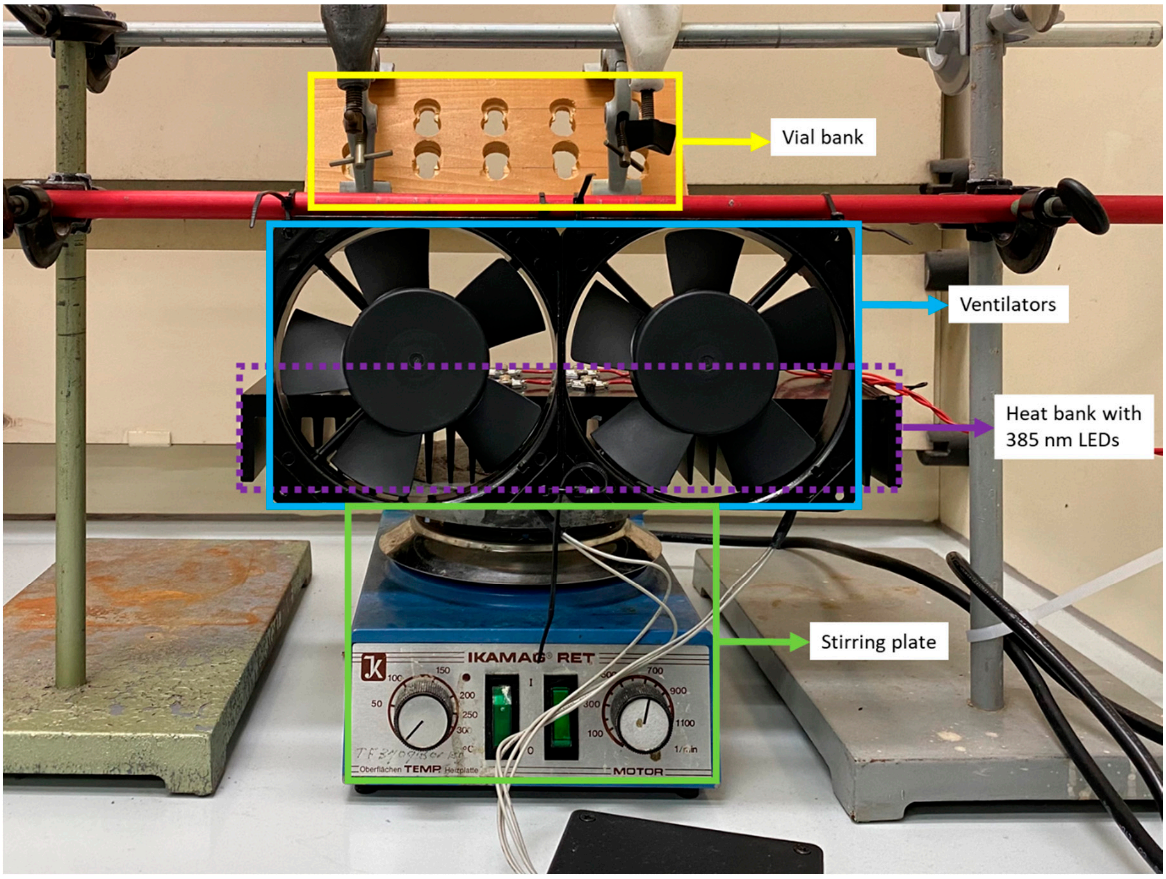

Figure S11: Irradiation set up consisting of a heat bank with 10x 385 nm LED, stirring plate, moveable vial bank suitable for 10x 1.5 mL vial and two ventilators to supply cool air to the samples.

Reaction mechanisms for the dehalogenation of aromatic halides

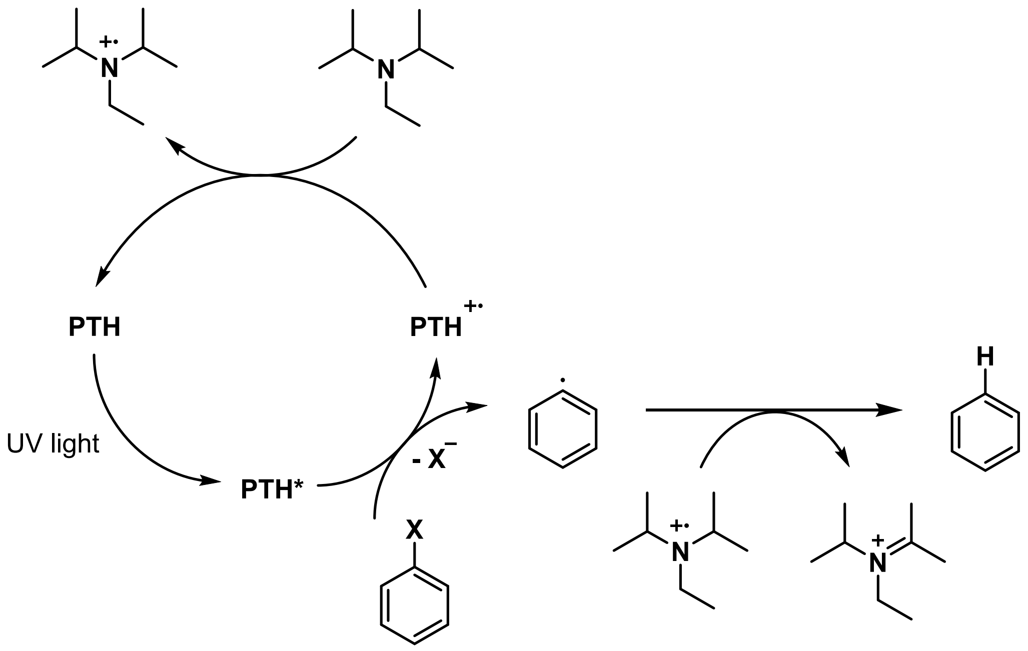

Figure S12: Proposed mechanism for metal-free dehalogenation of aromatic halides with phenothiazine as the photoredox catalyst and DIPEA as the tertiary amine base by Discekici et al [5].

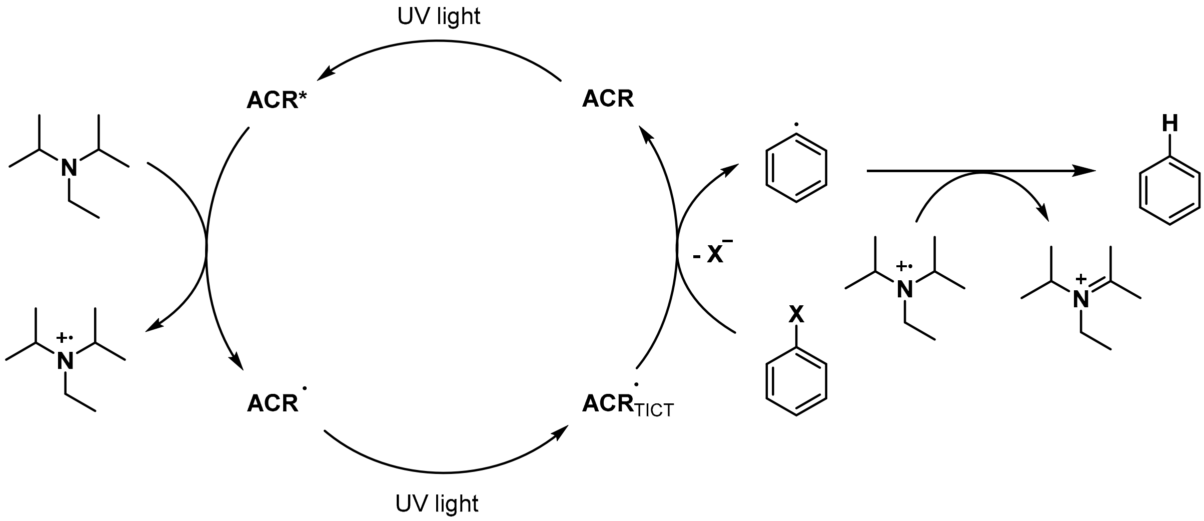

Figure S13: Proposed mechanism for metal-free dehalogenation of aromatic halides with acridinium as the photoredox catalyst and DIPEA as the tertiary amine base by MacKenzie et al [6].

Procedure *para*-substituted aryl halide reaction

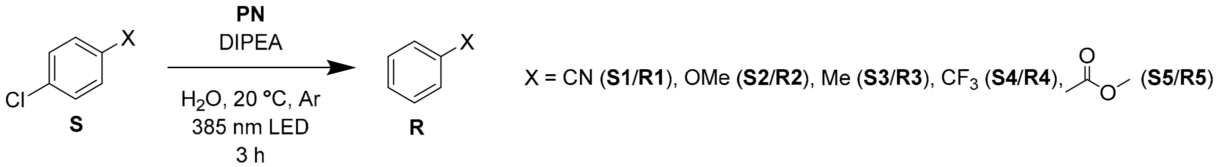

For the *para*-substituted aryl halide, 5 different substrates were tested. A solution of 10 mg of functionalized polymer in 0.5 mL of demineralized water was prepared. To this solution, the substrate (50 µmol, 1.0 eq.), DIPEA (43.7 µL, 250 µmol, 5.0 eq.) and a magnetic stirrer were added. The sample was subsequently degassed for 5 minutes with argon. The top of the vial was wrapped with Parafilm to make the vial as airtight as possible. The degassed sample was placed in the irradiation set up for 3 or 18 h, depending on the experiment. After the catalytic reaction, the sample was extracted 3 times with 0.5 mL of $CDCl_3$. Conversion was then measured by ^1H NMR spectroscopy, the estimated measurement error is ± 2%.

Conversion 4-chlorobenzonitrile (S1)

By comparing the ^{1}H NMR spectrum of both 4-chlorobenzonitrile and benzonitrile, full conversion was achieved since all substrate peaks disappeared after illumination for 3 h for every functionalized polymer.

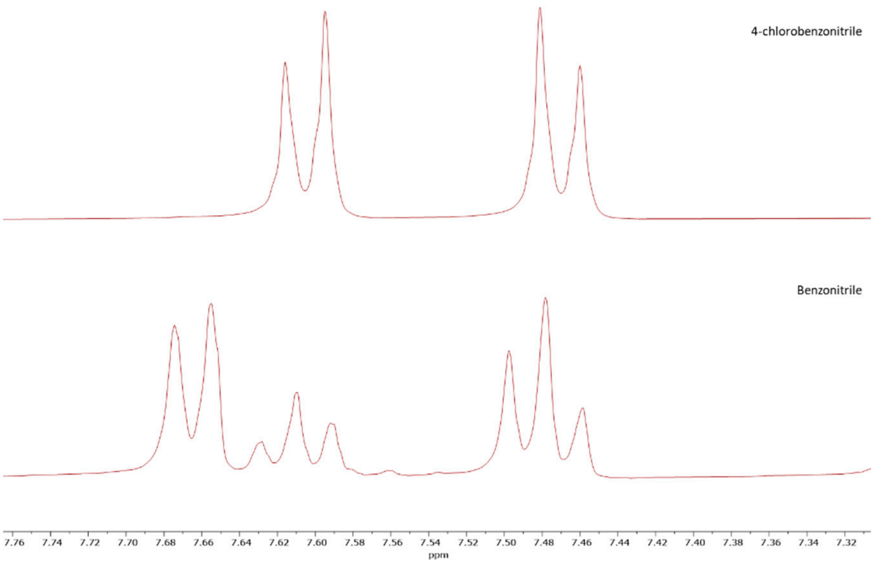

Figure S14: ^{1}H NMR spectra (400 MHz, $CDCl_3$, 25 °C) of pure 4-chlorobenzonitrile **S1** and full conversion to benzonitrile **R1** by **PN6**. Full conversion occurred for all six functionalized polymers.

Conversion 4-chloroanisole (S2)

The conversion of 4-chloroanisole **S2** to anisole **R2** is determined by comparing the ratio of the substrate doublet (2H) at 6.87 – 6.79 ppm and the product doublet (2H) and triplet (1H) at 6.98 – 6.89 ppm.

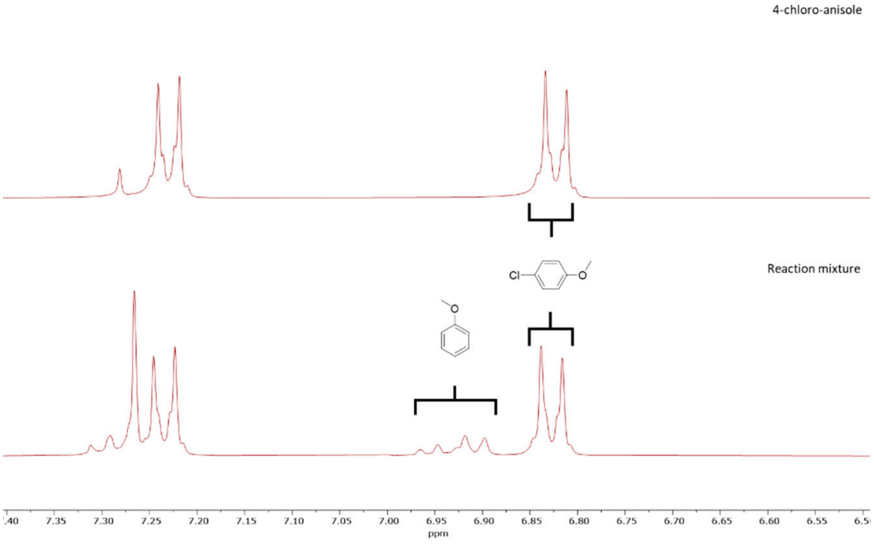

Figure S15: ^{1}H NMR spectra (400 MHz, $CDCl_3$, 25 °C) of pure 4-chloro-anisole **S2** and the reaction mixture containing product anisole **R2**.

Conversion 4-chlorotoluene (S3)

The conversion of 4-chlorotoluene **S3** to toluene **R3** is determined by comparing the ratio of the proton signal from the methyl group of the 4-chlorotoluene **S3** at 2.32 ppm and toluene **R3** at 2.35 ppm.

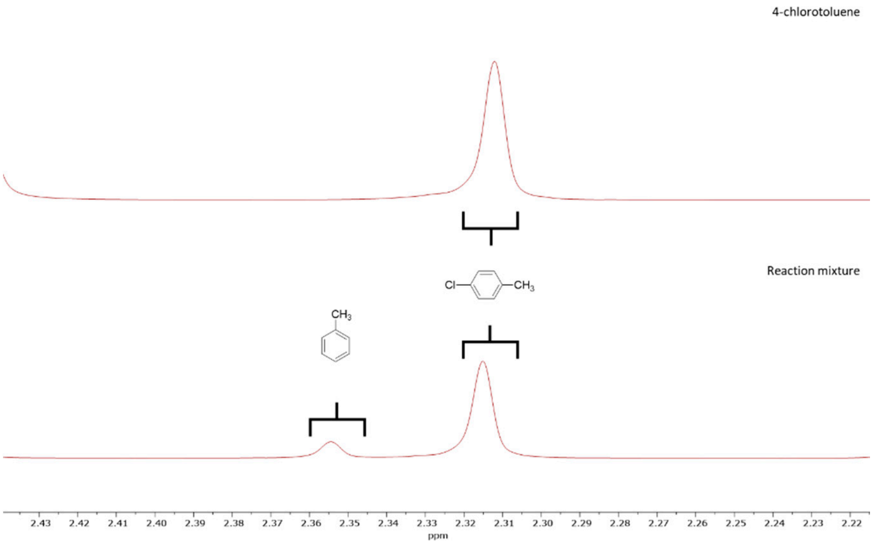

Figure S16: ^{1}H NMR spectra (400 MHz, $CDCl_3$, 25°C) of pure 4-chlorotoluene **S3** and the reaction mixture containing product toluene **R3**.

Conversion 4-chlorobenzotrifluoride (S4)

The conversion of 4-chlorobenzotrifluoride **S4** to benzotrifluoride **R4** is determined by comparing the ratio of the substrate doublet (2H) at 7.58 ppm and the product doublet (2H) at 7.63 ppm.

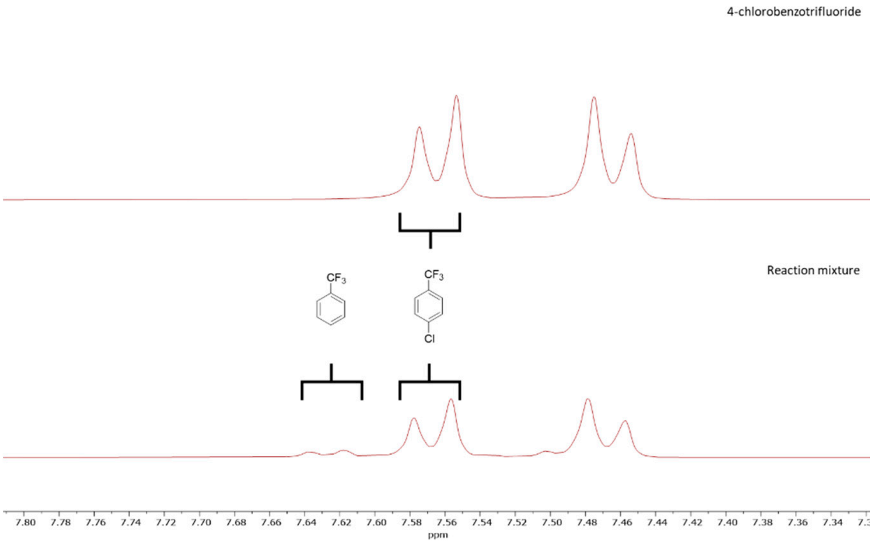

Figure S17: ^{1}H NMR spectra (400 MHz, $CDCl_3$, 25 °C) of pure 4-chlorobenzotrifluoride **S4** and the reaction mixture containing product benzotrifluoride **R4**.

Conversion methyl-4-chlorobenzoate (S5)

The conversion of methyl 4-chlorobenzoate **S5**to methyl benzoate **R5** is determined by comparing the ratio of the substrate doublet (2H) at 7.98 ppm and the product doublet (2H) at 8.04 ppm. Conversion of methyl 4-chlorobenzoate **S5** after 18 h is shown in figure **S20**.

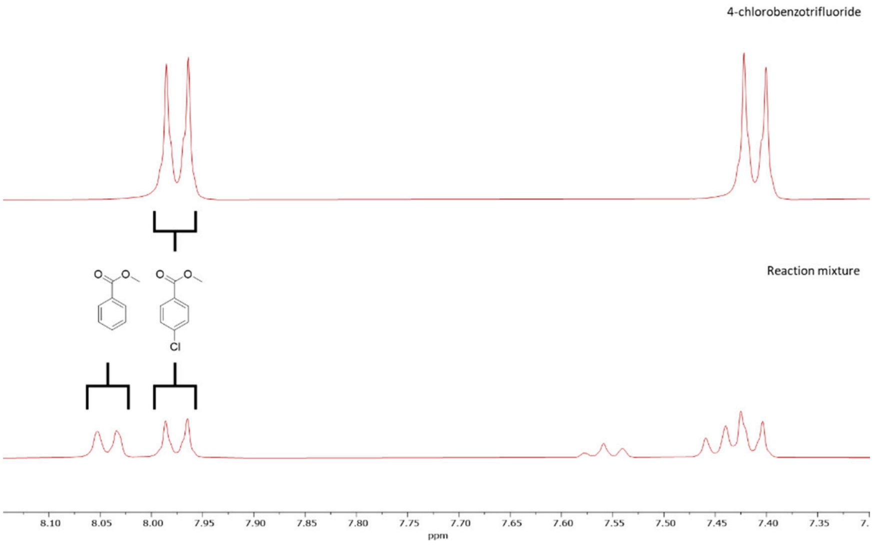

Figure S18: ^{1}H NMR spectra (400 MHz, $CDCl_3$, 25 °C) of pure methyl 4-chlorobenzoate **S5** and the reaction mixture containing product methyl benzoate **R5**.

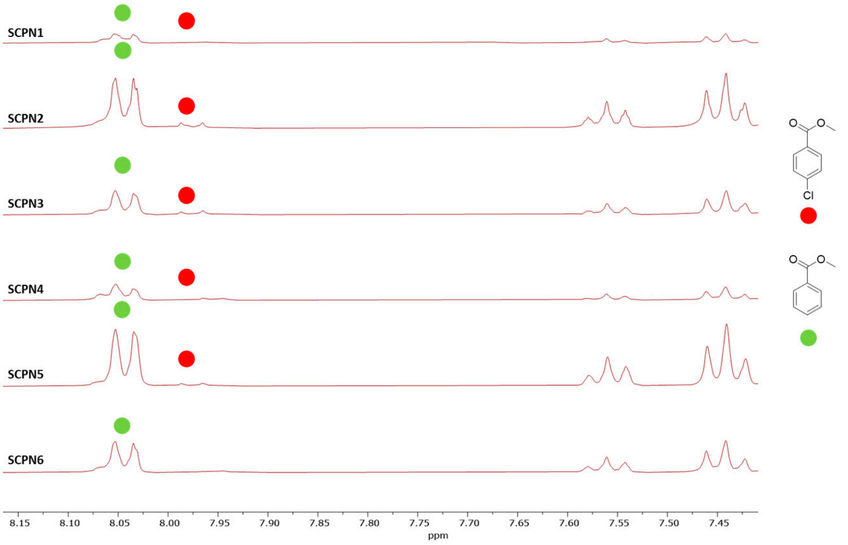

Figure S19: ^{1}H NMR spectra (400 MHz, $CDCl_3$, 25 °C) of pure methyl 4-chlorobenzoate **S5** and the reaction mixture of **PN1-6** in which part of the methyl 4-chlorobenzoate **S5** has been converted to methyl benzoate **R5** after 18 h of irradiation. Conversion for **PN1-PN6** is 98, 96, 93, 90, 97, and 96%, respectively.

Procedure selectivity reaction

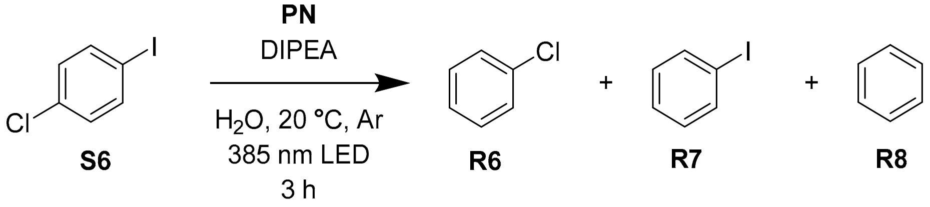

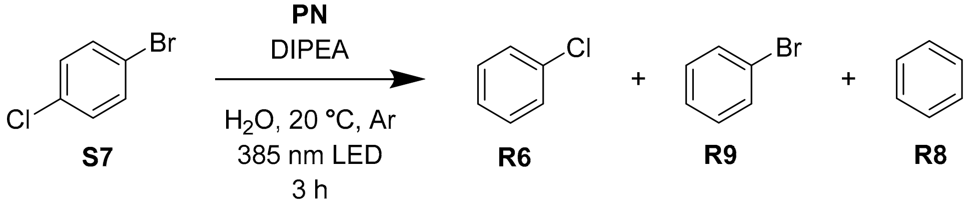

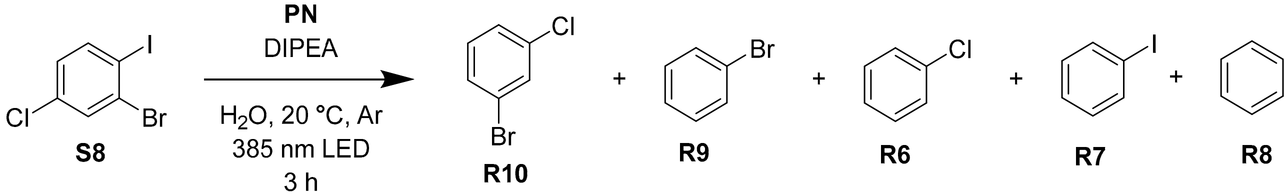

For the selectivity reactions, three different substrates were tested. A solution of 10 mg of functionalized polymer in 0.5 mL of distilled water was prepared. To this solution the substrate (50 µmol, 1 eq.) and DIPEA (43.7 µL, 250 µmol, 5 eq.). The top of the vial was wrapped with Parafilm to make the vial as airtight as possible. The sample was subsequently degassed for 5 minutes with argon. The degassed sample was placed in the irradiation set up for 3 or 24 h, depending on the experiment. After the catalytic reaction, the sample was extracted 3 times with 0.5 mL of $CDCl_3$. Conversion was then measured by ^1H NMR spectroscopy.

Conversion 1-chloro-4-iodobenzene (S6)

The conversion of 1-chloro-4-iodobenzene **S6** to chlorobenzene **R6** and benzene **R8** was determined with the aid of deconvolution. Since the signals of chlorobenzene (δ = 7.36 – 7.24 ppm) and benzene (δ = 7.36 ppm) overlap, deconvolution is used to determine the area of the benzene peak. Besides this, deconvolution is also used to remove the $CDCl_3$ peak at δ = 7.26 ppm.

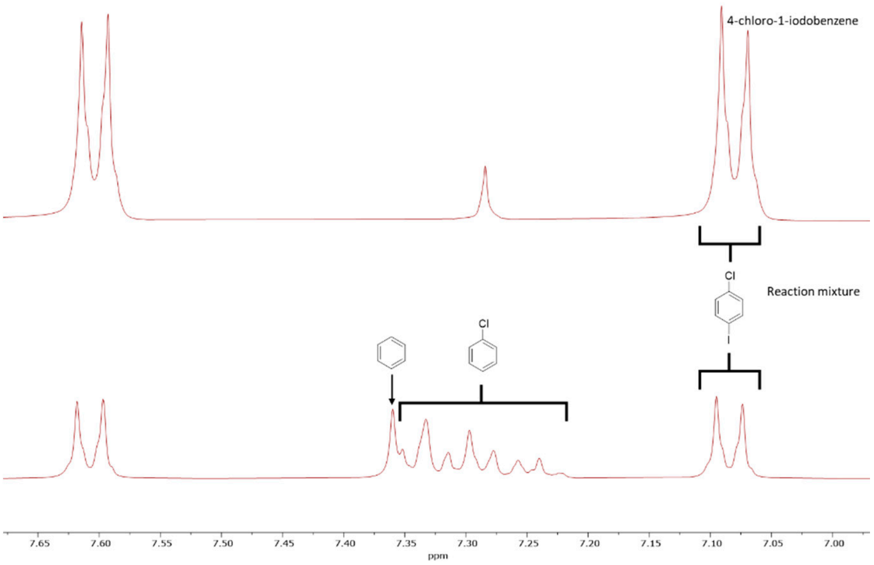

Figure S20: ^{1}H NMR spectra (400 MHz, $CDCl_3$, 25 °C) of pure 4-chloro-1-iodobenzene **S6** and the reaction mixture in which part of the 4-chloro-1-iodobenzene **S6** has been converted into chlorobenzene **R6** and benzene **R8**.

Conversion 1-bromo-4-chlorobenzene (S7)

The conversion of 1-bromo-4-chlorobenzene **S7** to chlorobenzene **R6** and benzene **R8** was determined with the aid of deconvolution. Since the signals of chlorobenzene (δ = 7.36 – 7.24 ppm) and benzene (δ = 7.36 ppm) overlap, deconvolution was applied to determine the intensity of the benzene peak. The doublet of the chlorobenzene at 7.35 ppm (2H) is compared to the doublet of 1-bromo-4-chlorobenzene **S7** at 7.42 ppm.

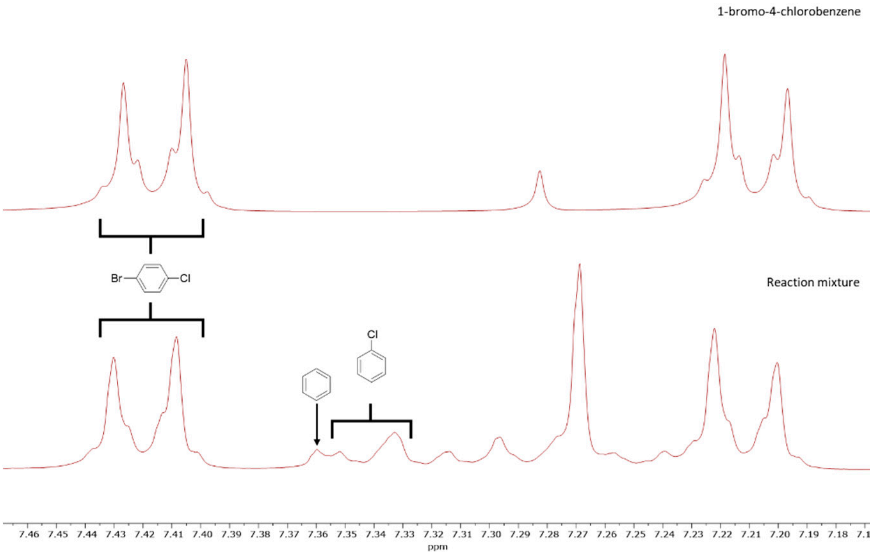

Figure S21: ^{1}H NMR spectra (400 MHz, $CDCl_3$, 25 °C) of pure 1-bromo-4-chlorobenzene **S7** and the reaction mixture in which part of the 1-bromo-4-chlorobenzene **S7** was converted to chlorobenzene **R6** and benzene **R8**.

Qualitative analysis 2-bromo-4-chloro-1-iodobenzene (S8)

Since the signals of the 1-bromo-3-chlorobenzene **R10** and chlorobenzene **R6** overlap, only a qualitative analysis of the photocatalytic reaction with 2-bromo-4-chloro-1-iodobenzene was performed. ^{1}H NMR spectra of the reaction products **R6**, **R8**, **R9**, **R10**[1] and **R11** are compared to the ^{1}H NMR spectrum of the reaction mixture.

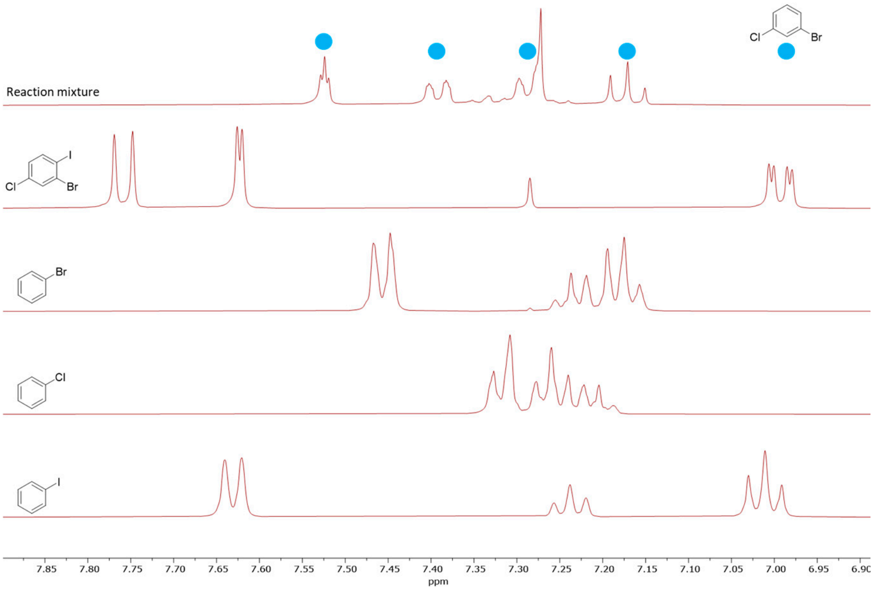

Figure S22: ^{1}H NMR spectra (400 MHz, $CDCl_3$, 25 °C) of reaction mixture, substrate 2-bromo-4-chloro-1-iodobenzene **S8** and possible reaction products.

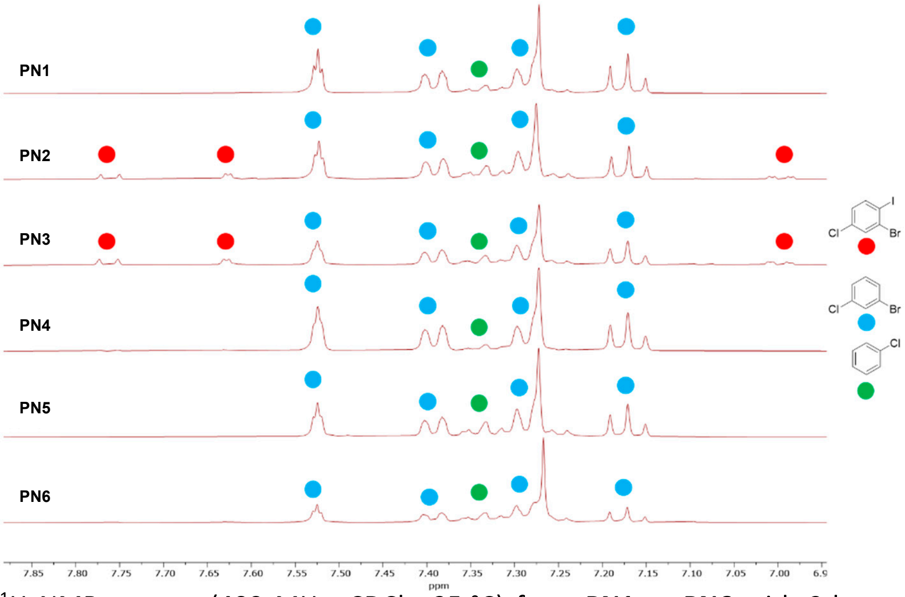

Figure S23: ^{1}H NMR spectra (400 MHz, $CDCl_3$, 25 °C) from **PN1** to **PN6** with 2-bromo-4-chloro-1-iodobenzene **S8** as substrate after 3 h. The peaks are marked with colored circles. Red circles correspond to 2-bromo-4-chloro-1-iodobenzene **S8**, blue to 1-bromo-3-chlorobenzene **R10,** and green to chlorobenzene **R6**.

[1] The ^{1}H NMR spectrum of 1-bromo-3-chlorobenzene R10 is obtained from literature.[7]

Procedure for C-C cross coupling reactions

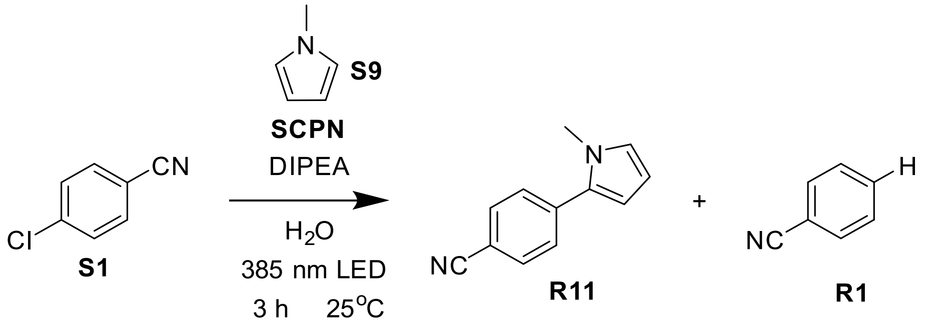

A solution of 10 mg of functionalized polymer was dissolved in 0.5 mL of distilled water. To this solution, 4-chloro-benzonitrile **S1** (6.9 mg, 50 µmol, 1 eq.), *N*-methyl pyrrole **S9** (22.2 µL, 250 µmol, 5 eq.) and DIPEA (43.7 µL, 250 µmol, 5 eq.). The top of the vial was wrapped with Parafilm to make it as airtight as possible. The sample was subsequently degassed for 5 minutes with argon. The degassed sample was placed in the irradiation set up for 3 h. Afterwards, the sample was extracted three times with 0.5 mL of $CDCl_3$. Conversion was measured by ^1H NMR spectroscopy.

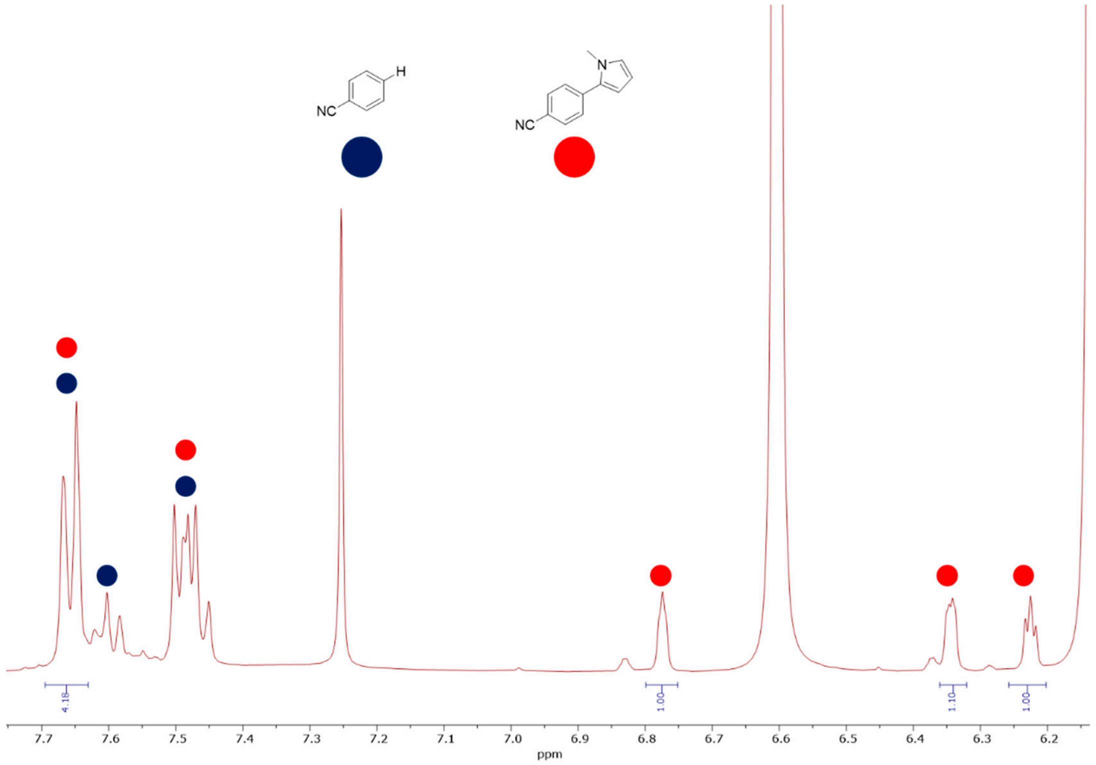

Figure S24: ^1H NMR spectrum (400 MHz, $CDCl_3$, 25 °C) after C-C cross coupling reaction mixture with assigned peaks. The doublet at 7.66 ppm corresponds to both 2 protons on the protonated and C-C cross coupled product. Conversion of 4-chlorobenzonitrile **S1** was observed to be quantitative for all functionalized polymers.

References

1. Choi, J.; Schattling, P.; Jochum, F.D.; Pyun, J.; Char, K.; Theato, P. Functionalization and patterning of reactive polymer brushes based on surface reversible addition and fragmentation chain transfer polymerization. *J. Polym. Sci. Part A Polym. Chem.* **2012**, *50*, 4010–4018, doi:10.1002/pola.26200.

2. Eisenreich, F.; Meijer, E.W.; Palmans, A.R.A. Amphiphilic Polymeric Nanoparticles for Photoredox Catalysis in Water. *Chem. Eur. J.* **2020**, *26*, 10355–10361, doi:10.1002/chem.202001767.

3. White, A.; Wang, L.; Nicewicz, D. Synthesis and Characterization of Acridinium Dyes for Photoredox Catalysis. *Synlett* **2019**, *30*, 827–832, doi:10.1055/s-0037-1611744.

4. Steiner, A.; Williams, J.D.; Rincón, J.A.; de Frutos, O.; Mateos, C.; Kappe, C.O. Implementing Hydrogen Atom Transfer (HAT) Catalysis for Rapid and Selective Reductive Photoredox Transformations in Continuous Flow. *European J. Org. Chem.* **2019**, *2019*, 5807–5811, doi:10.1002/ejoc.201900952.

5. Discekici, E.H.; Treat, N.J.; Poelma, S.O.; Mattson, K.M.; Hudson, Z.M.; Luo, Y.; Hawker, C.J.; de Alaniz, J.R. A highly reducing metal-free photoredox catalyst: design and application in radical dehalogenations. *Chem. Commun.* **2015**, *51*, 11705–11708, doi:10.1039/C5CC04677G.

6. MacKenzie, I.A.; Wang, L.; Onuska, N.P.R.; Williams, O.F.; Begam, K.; Moran, A.M.; Dunietz, B.D.; Nicewicz, D.A. Discovery and characterization of an acridine radical photoreductant. *Nature* **2020**, *580*, 76–80, doi:10.1038/s41586-020-2131-1.

7. Kim, H.; Lee, C. Visible-light-induced photocatalytic reductive transformations of organohalides. *Angew. Chem. Int. Ed.* **2012**, *51*, 12303–12306, doi:10.1002/anie.201203599.

NMR spectra

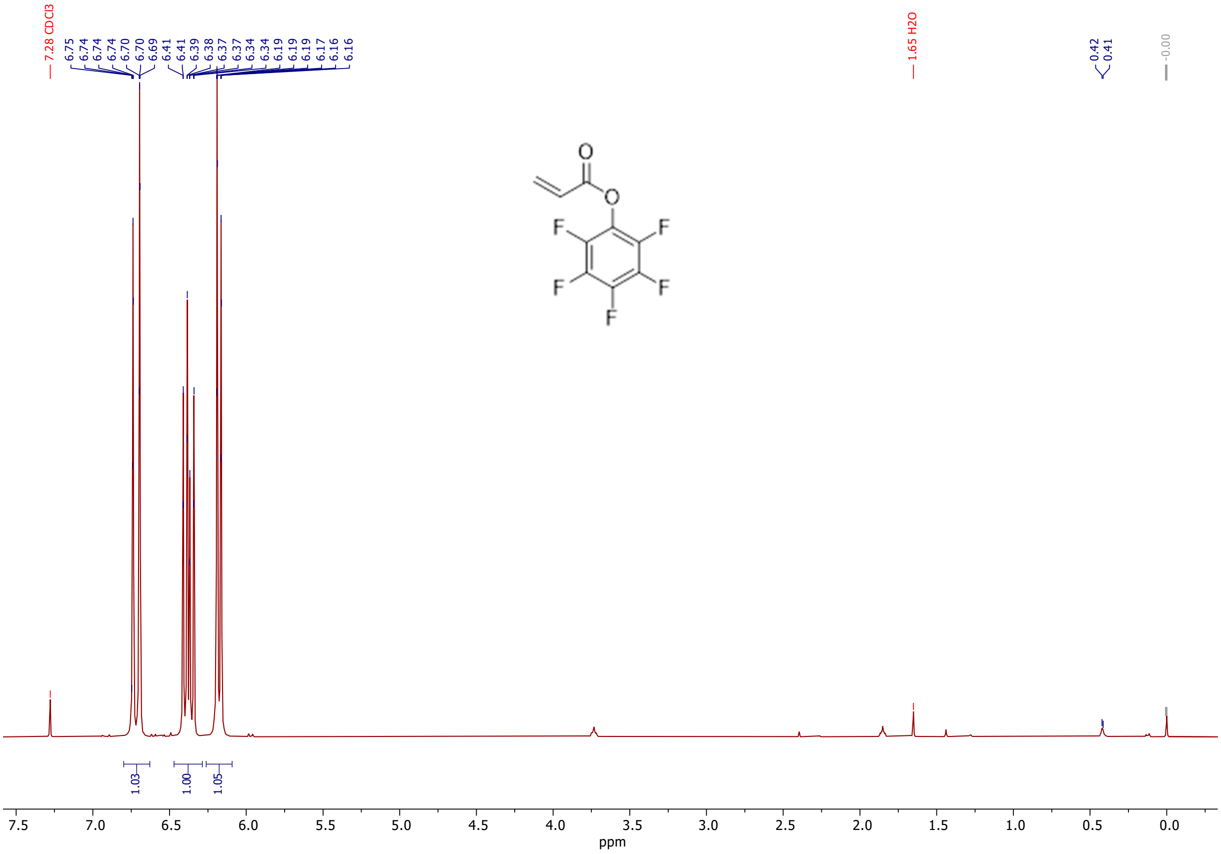

Figure S25: ^{1}H NMR spectrum (400 MHz, $CDCl_3$, 25 °C) of pentafluorophenyl acrylate **M1**.

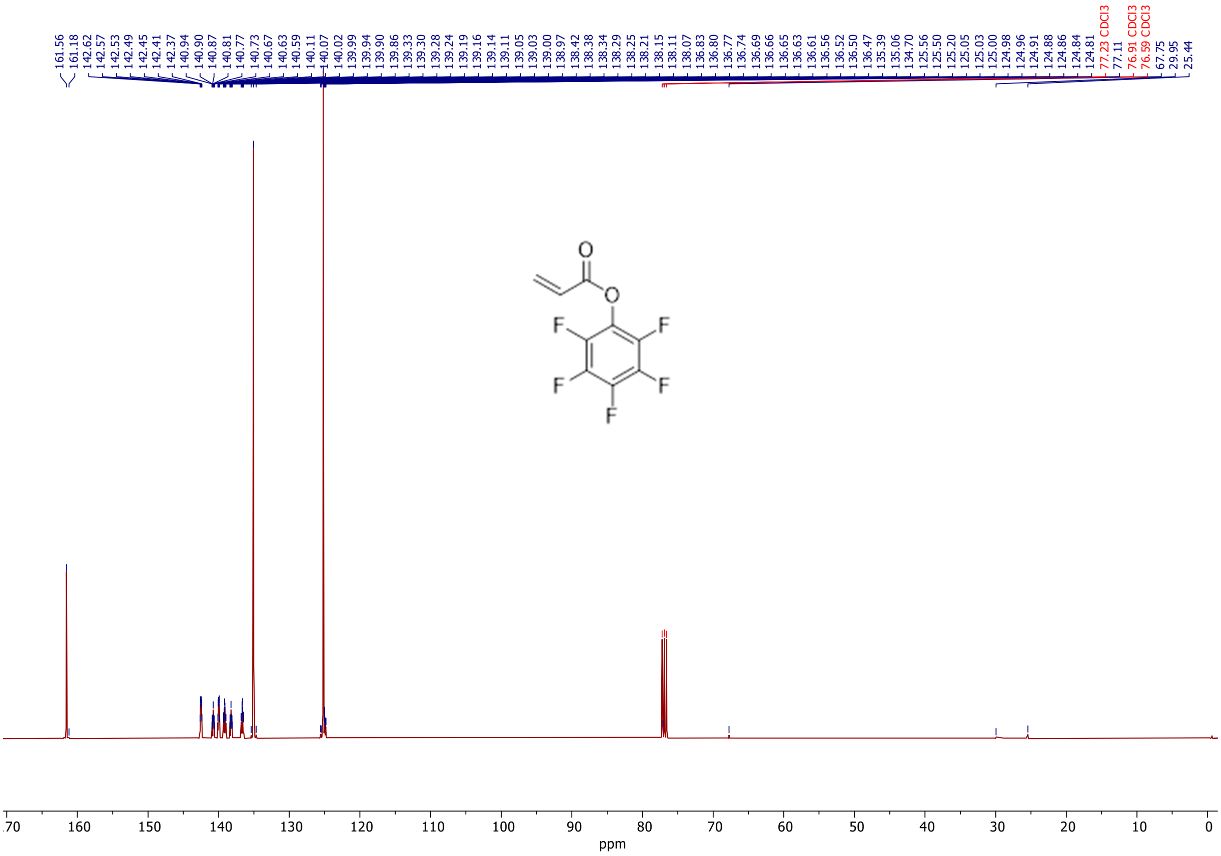

Figure S26: ^{13}C NMR spectrum (100 MHz, $CDCl_3$, 25 °C) of pentafluorophenyl acrylate **M1**.

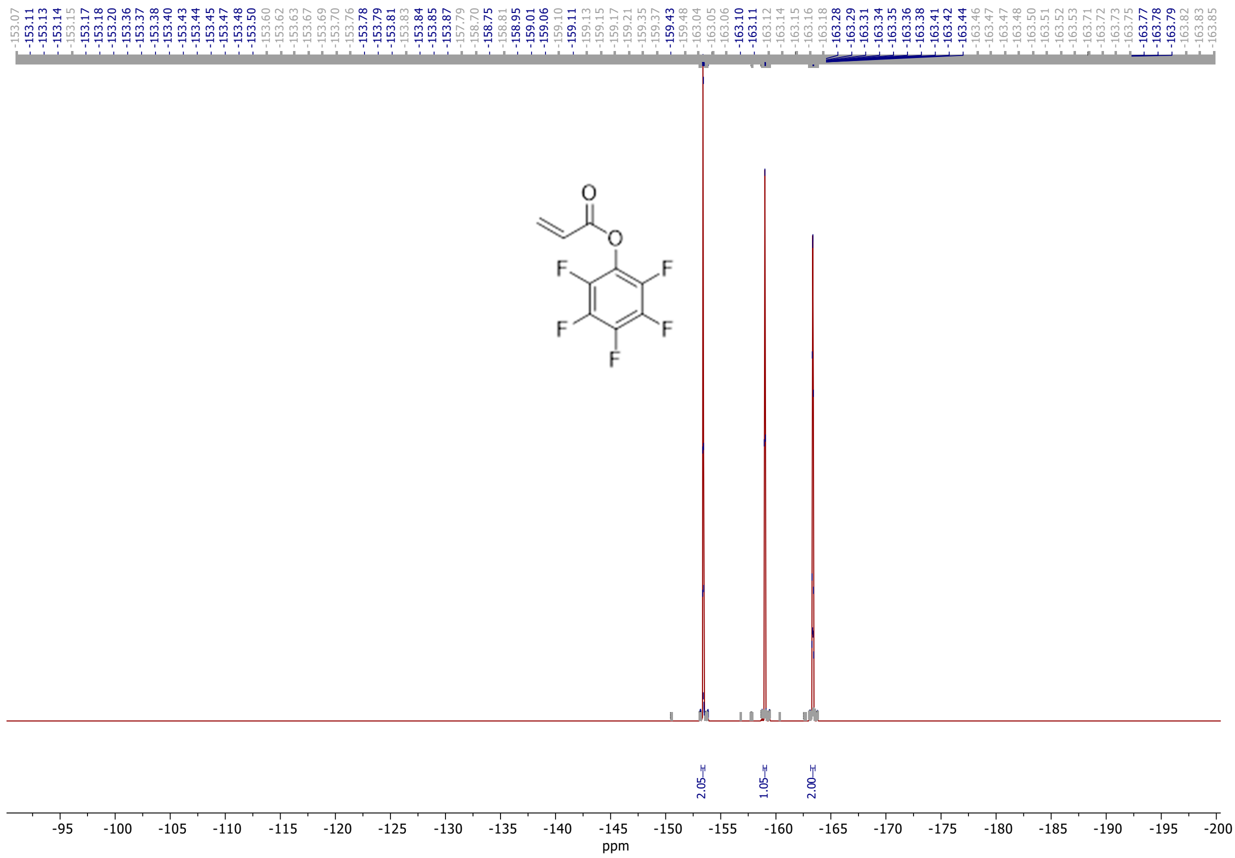

Figure S27: ^{19}F NMR spectrum (376 MHz, $CDCl_3$, 25 °C) of pentafluorophenyl acrylate **M1**.

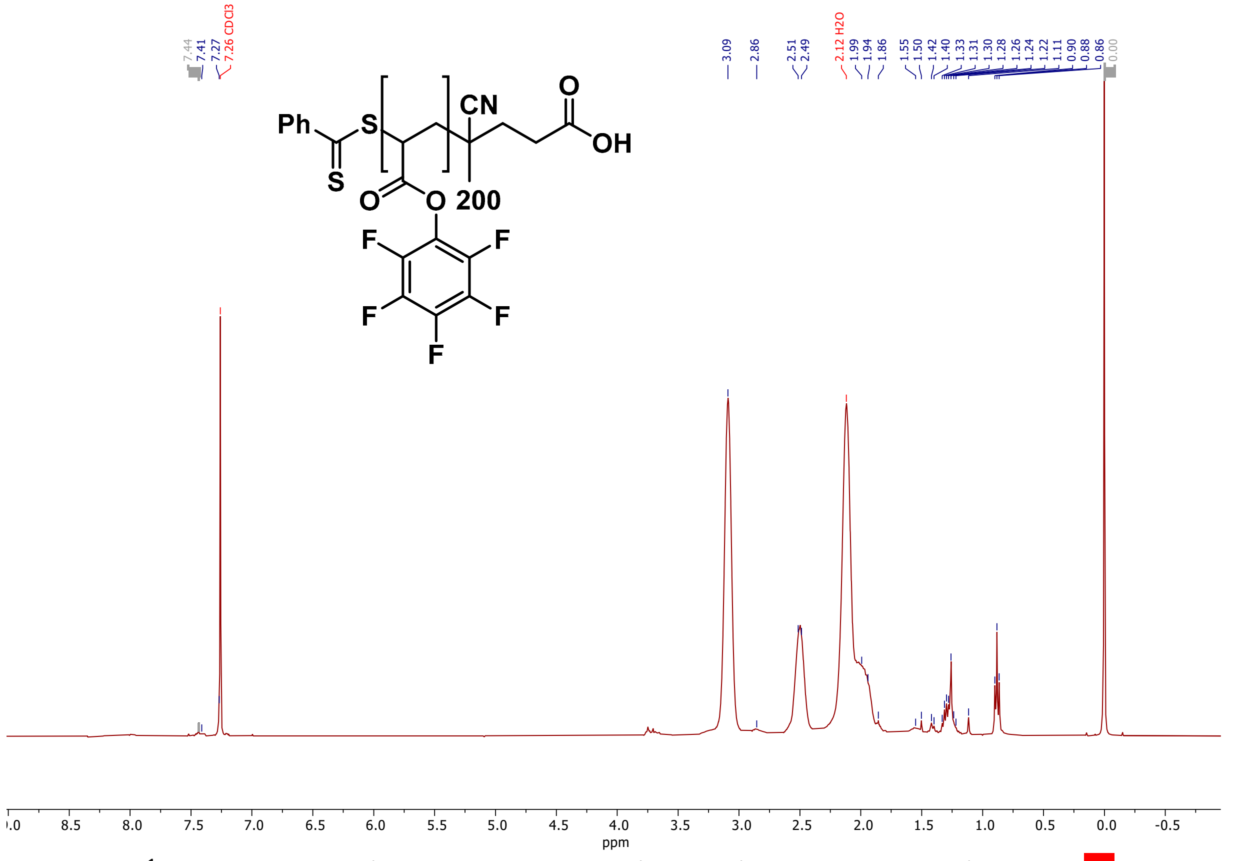

Figure S28: ^{1}H NMR spectrum (400 MHz, $CDCl_3$, 25 °C) of poly(pentafluorophenyl) acrylate **P1**.

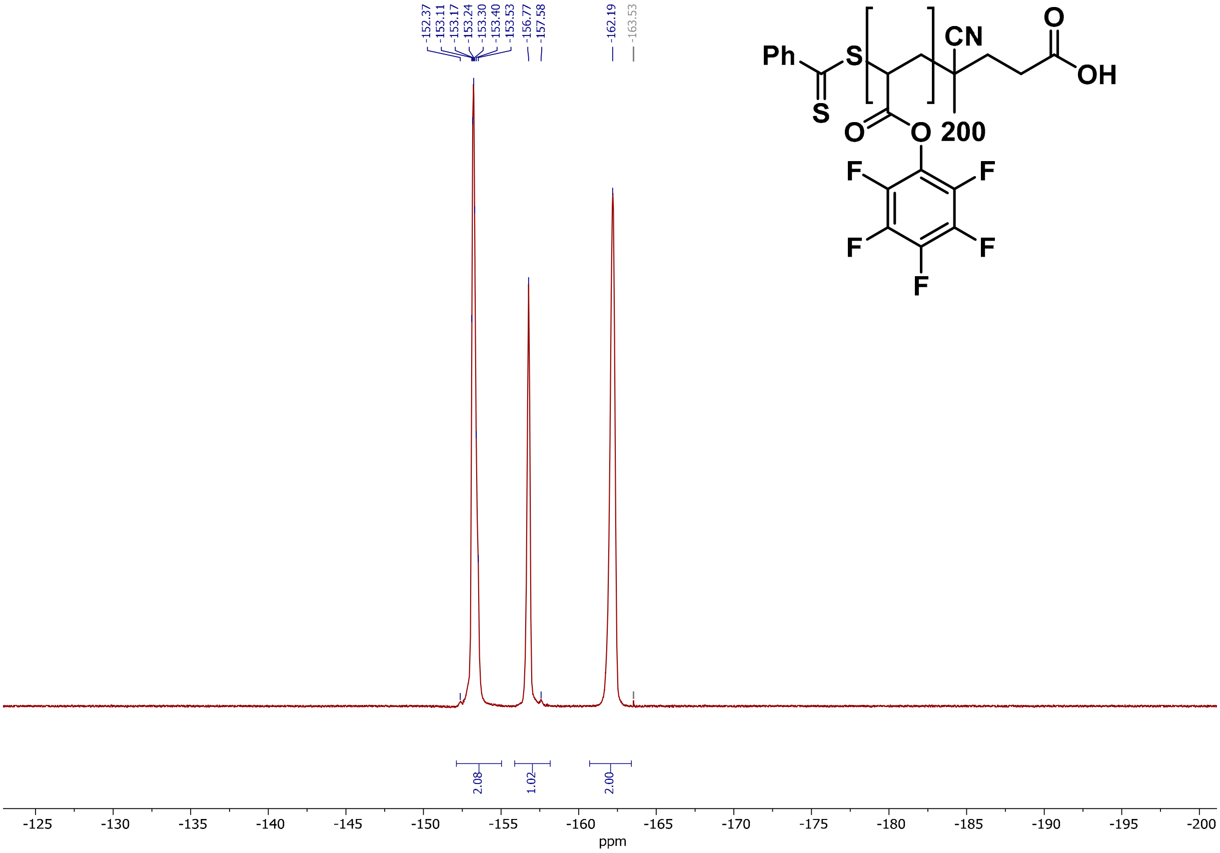

Figure S29: ^{19}F NMR spectrum (376 MHz, $CDCl_3$, 25 °C) of poly(pentafluorophenyl) acrylate **P1**.

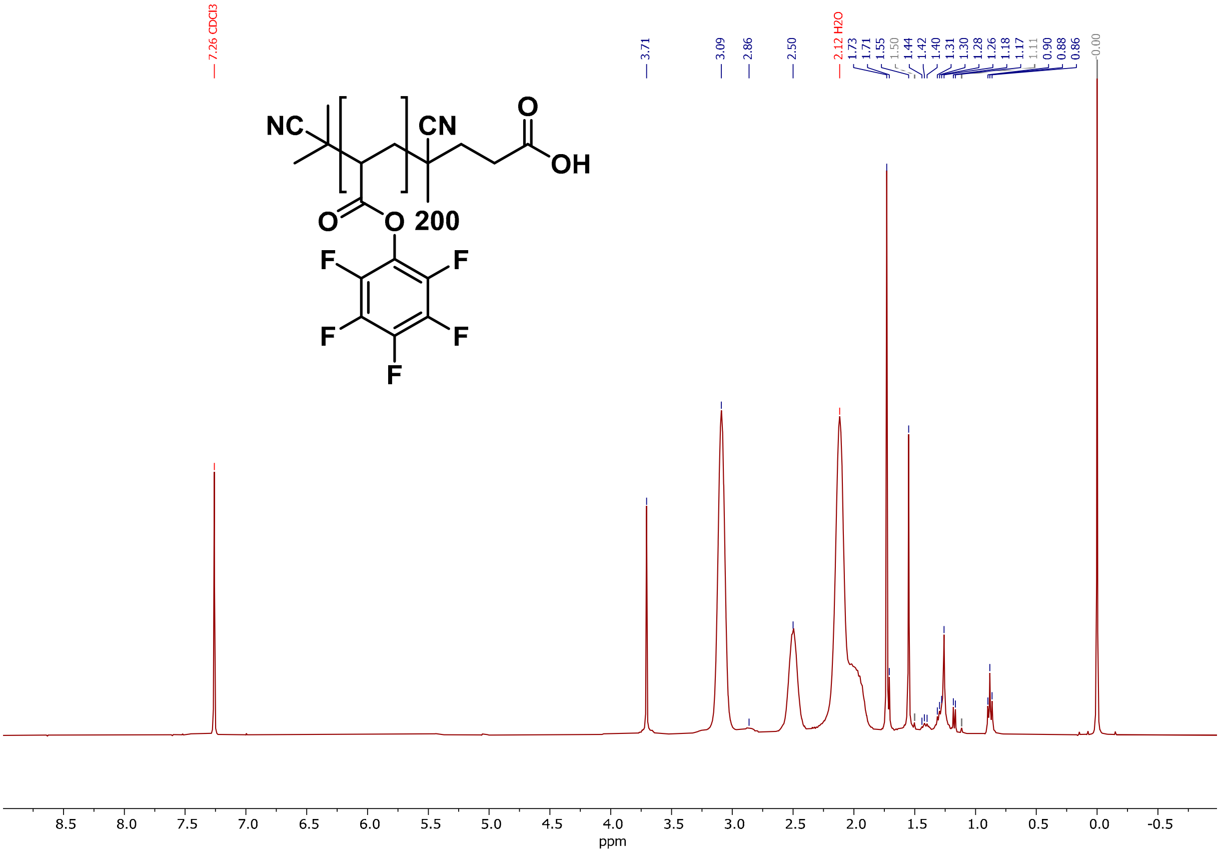

Figure S30: ^{1}H NMR spectrum (400 MHz, $CDCl_3$, 25 °C) of end-cap modified poly(pentafluorophenyl) acrylate **P2**.

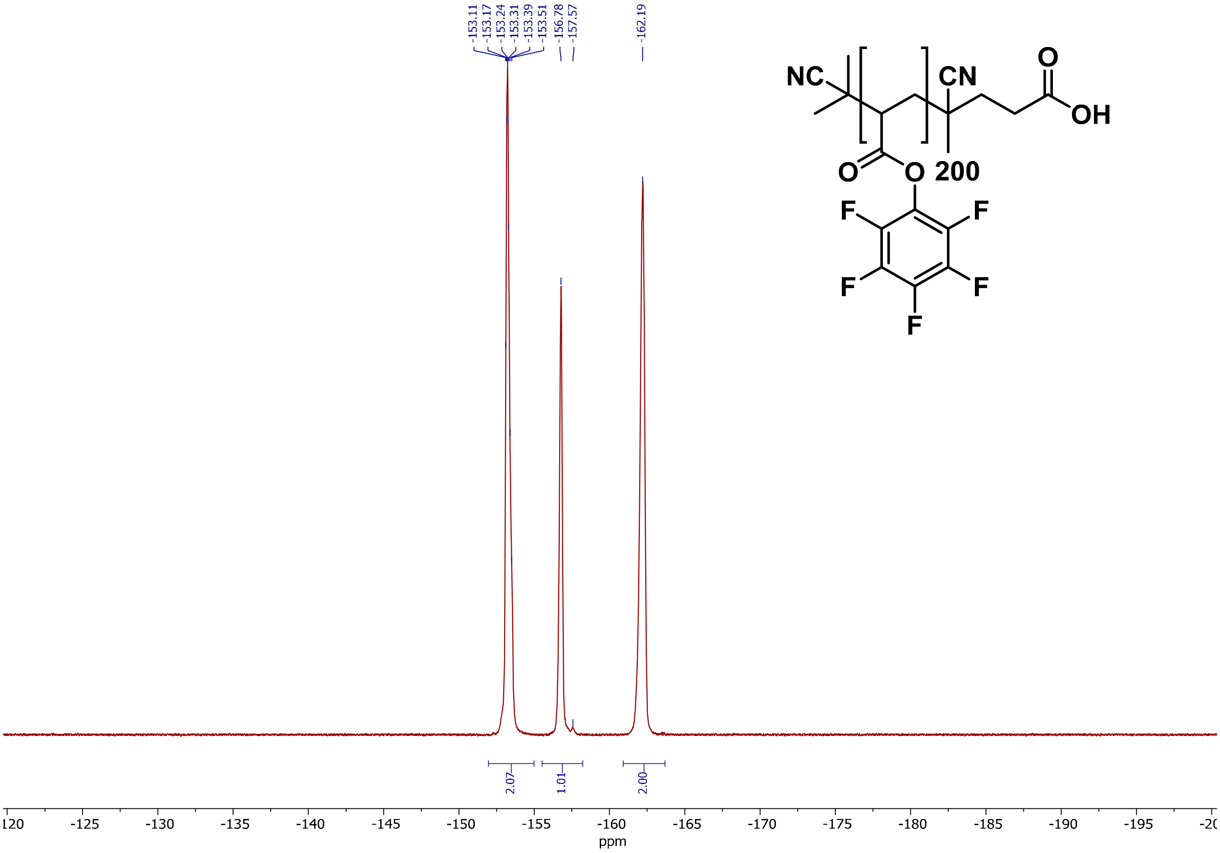

Figure S31: ^{19}F NMR spectrum (376 MHz, $CDCl_3$, 25 °C) of end-cap modified poly(pentafluorophenyl) acrylate **P2**.

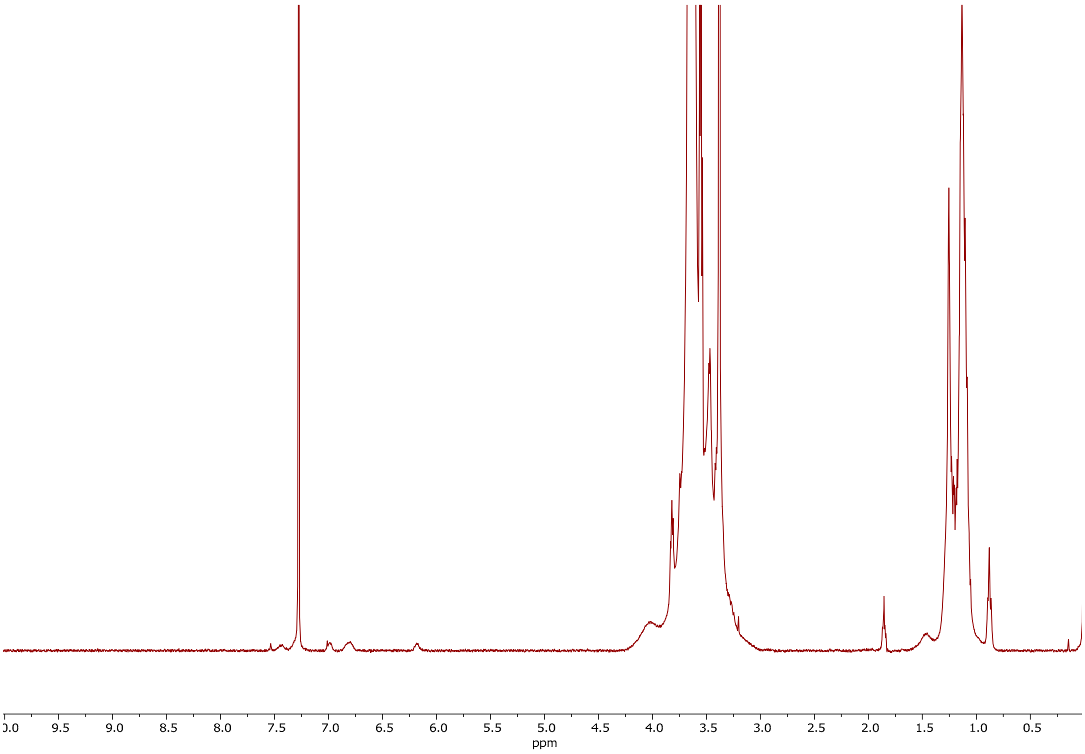

Figure S32: ^{1}H NMR spectrum (400 MHz, $CDCl_3$, 25 °C) of **PN1**. Signals of phenothiazine are located in aromatic region (δ = 6.0 – 7.5 ppm).

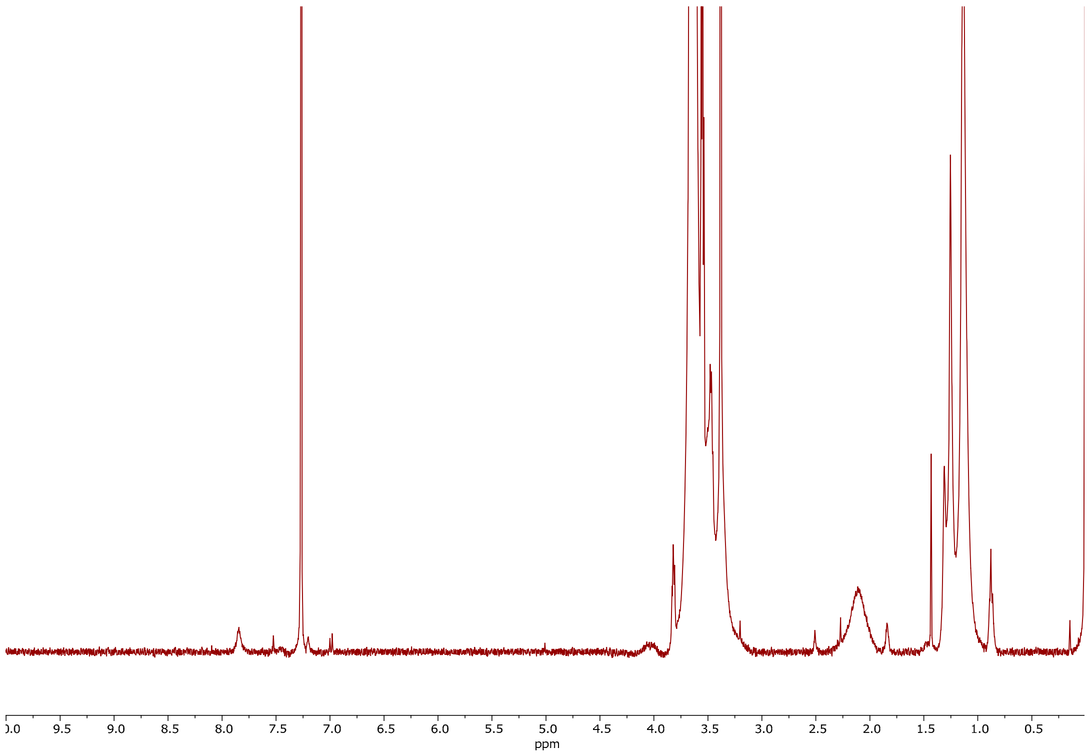

Figure S33: ^{1}H NMR spectrum (400 MHz, $CDCl_3$, 25 °C) of **PN2**. Signals of acridinium are located in aromatic region (δ = 6.0 – 8.0 ppm).

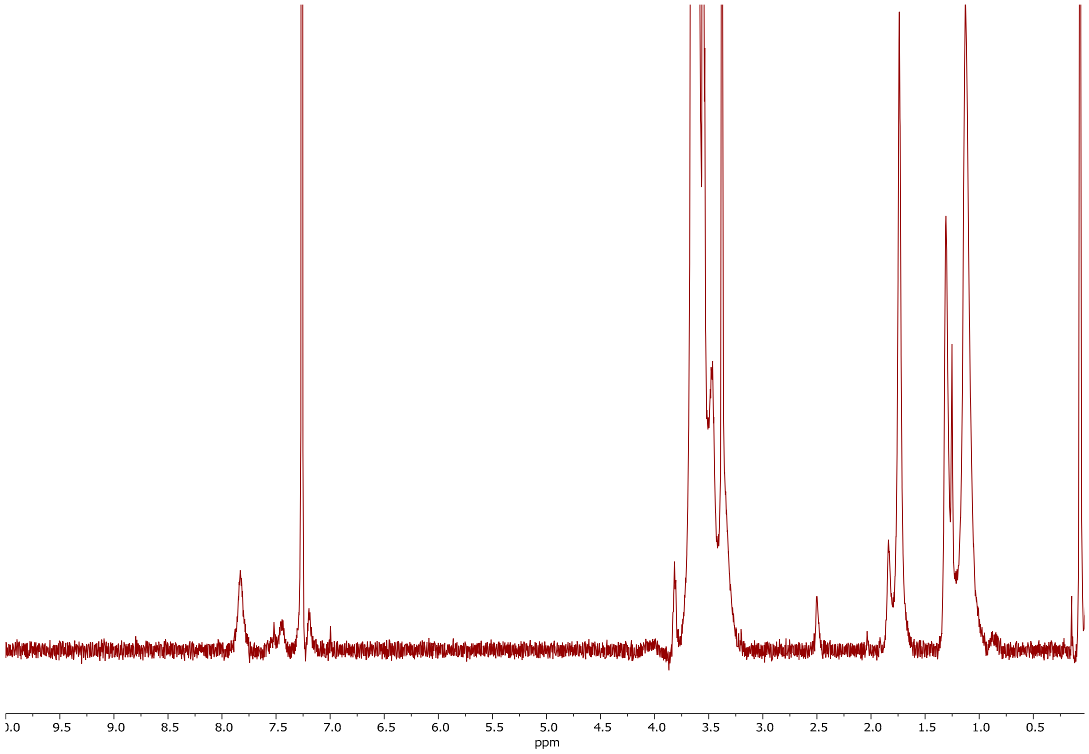

Figure S34: ^{1}H NMR spectrum (400 MHz, $CDCl_3$, 25 °C) of **PN3**. Signals of acridinium are located in aromatic region (δ = 6.0 – 8.0 ppm).

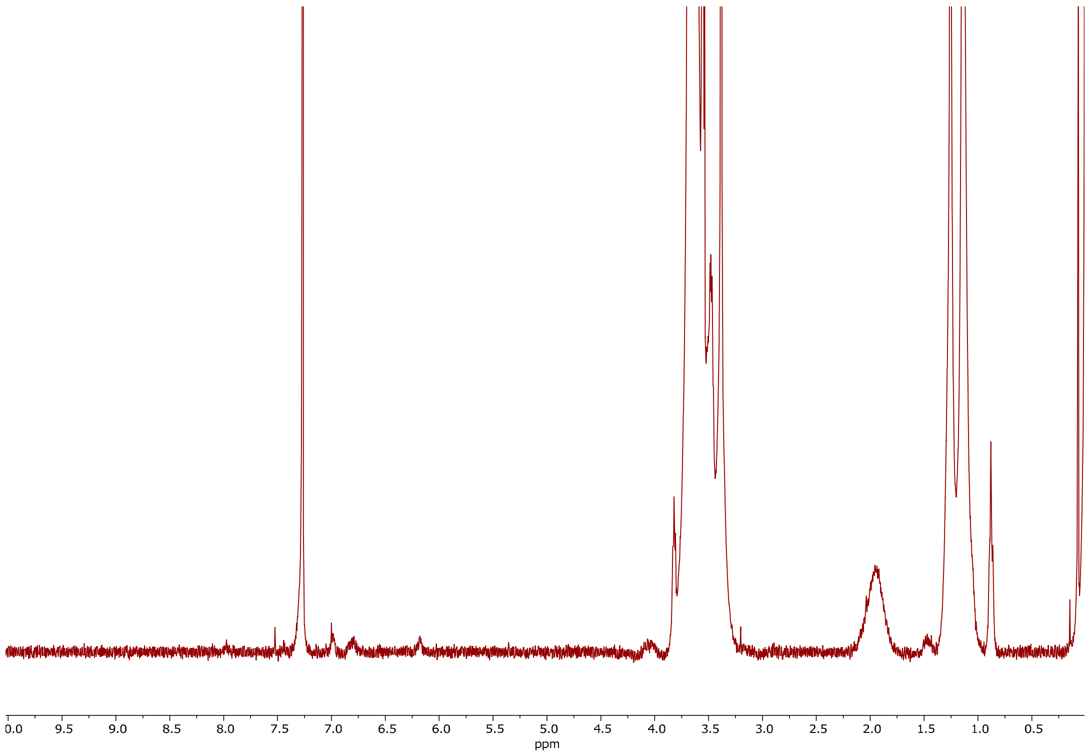

Figure S35: ^{1}H NMR spectrum (400 MHz, $CDCl_3$, 25 °C) of **PN4**. Signals of phenothiazine are located in aromatic region (δ = 6.0 – 7.5 ppm).

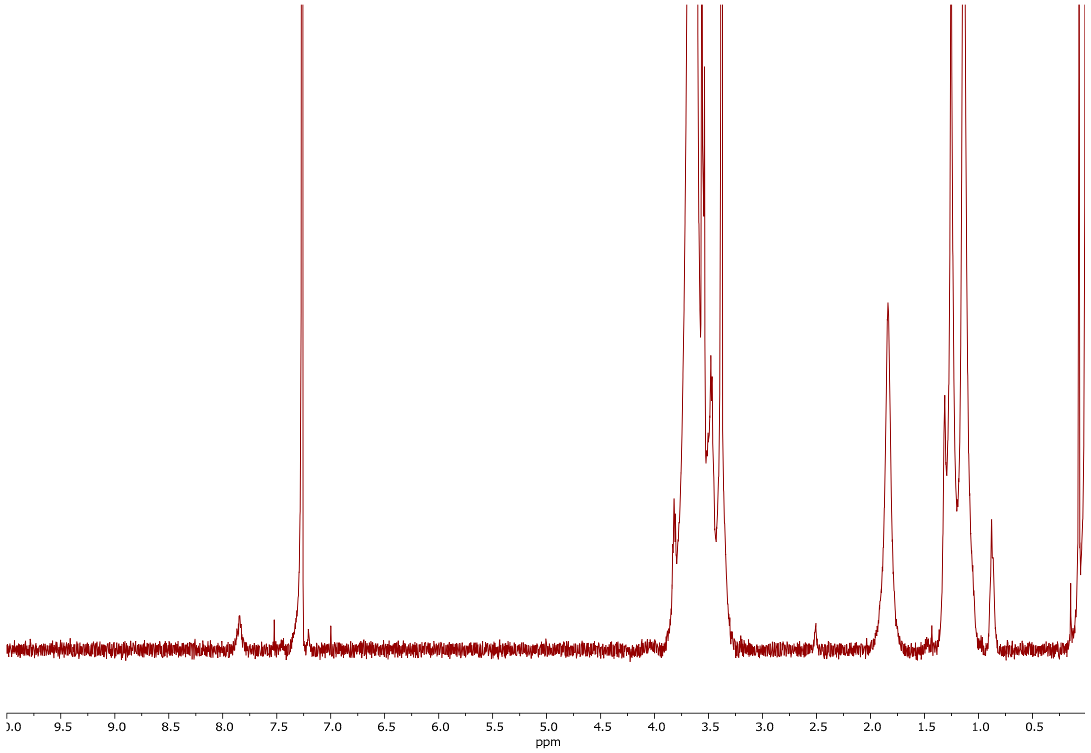

Figure S36: ^{1}H NMR spectrum (400 MHz, $CDCl_3$, 25 °C) of **PN5**. Signals of acridinium are located in aromatic region (δ = 6.0 – 8.0 ppm).

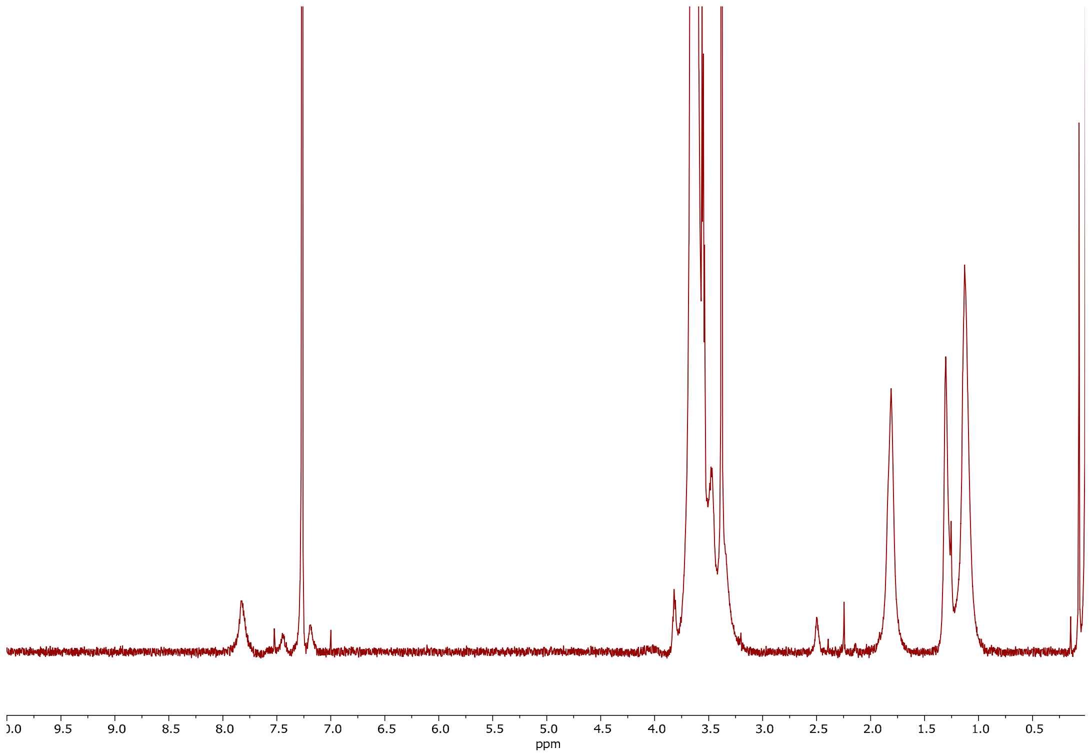

Figure S37: ^{1}H NMR spectrum (400 MHz, $CDCl_3$, 25 °C) of **PN6**. Signals of acridinium are located in aromatic region (δ = 6.0 – 8.0 ppm).

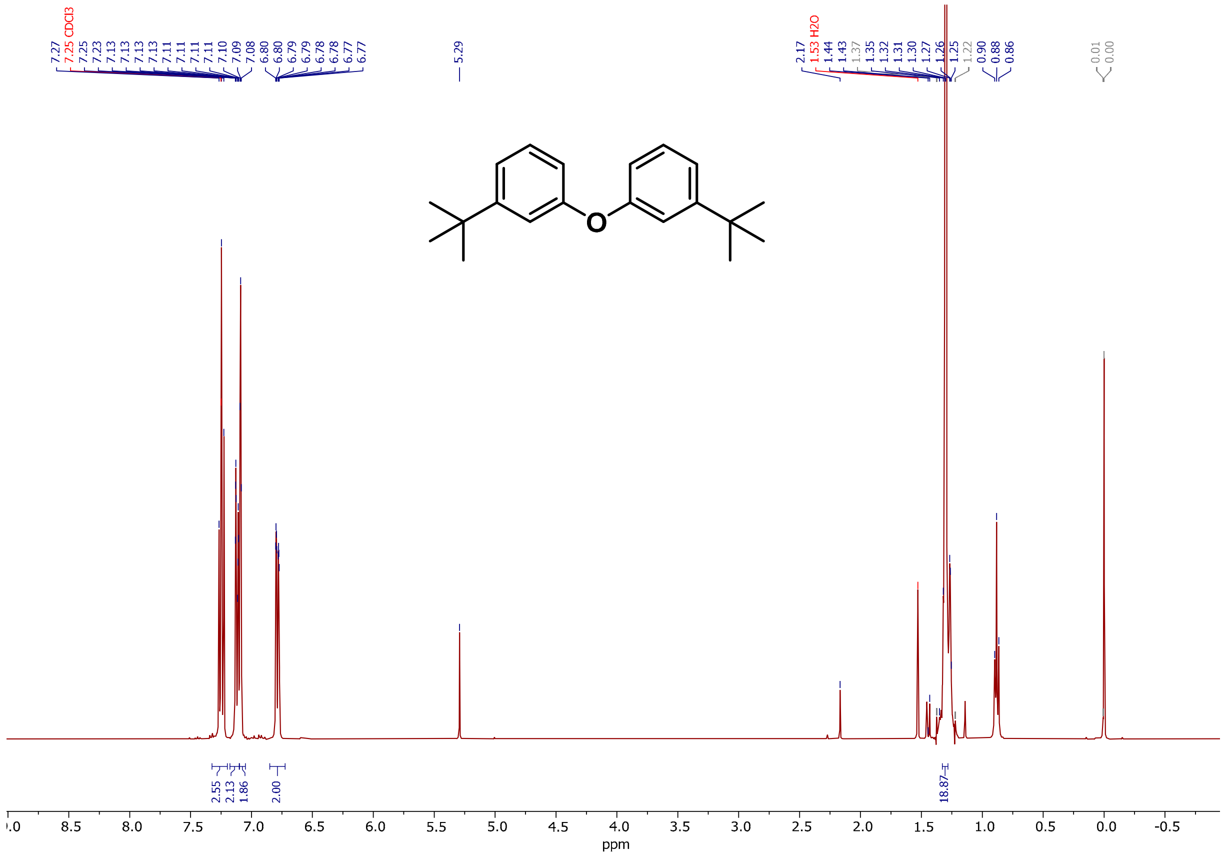

Figure S38: ^{1}H NMR spectrum (400 MHz, $CDCl_3$, 25 °C) of 3,3'-oxybis(*tert*-butylbenzene) **A1**.

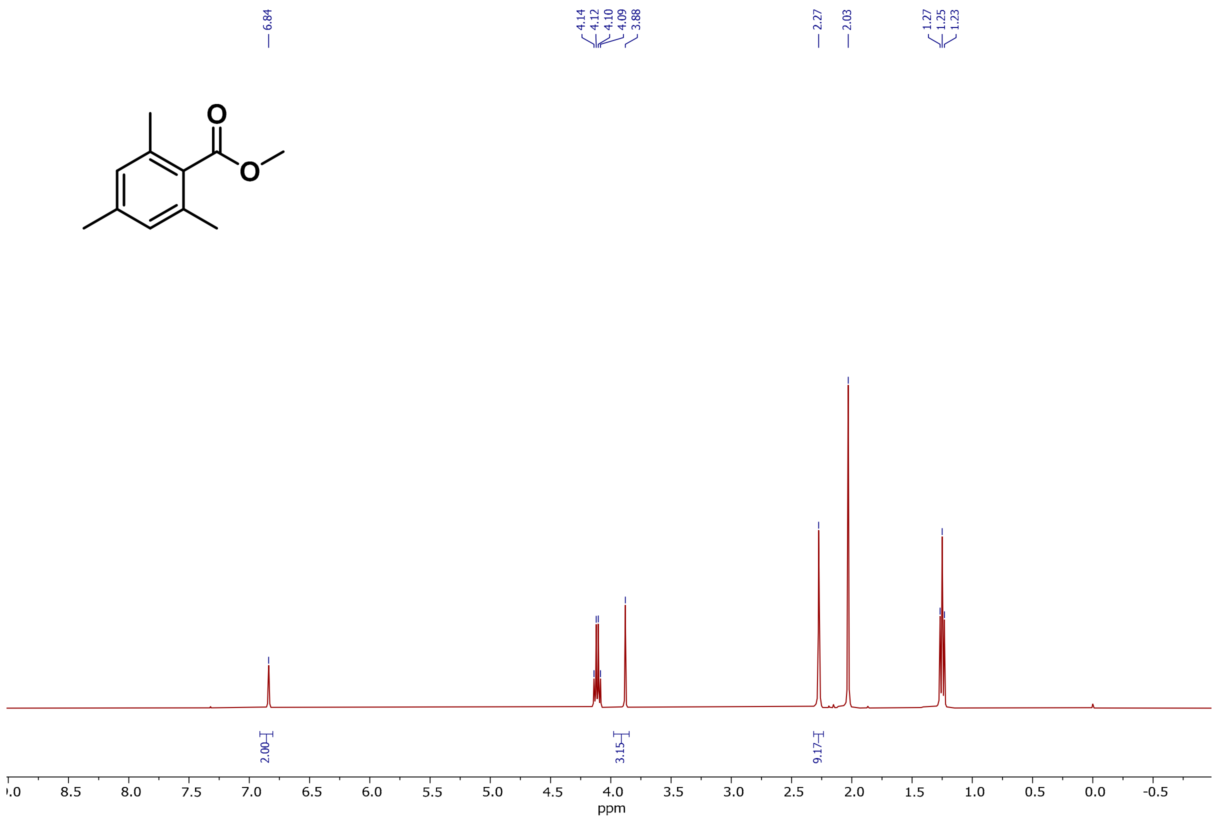

Figure S39: ^{1}H NMR spectrum (400 MHz, $CDCl_3$, 25 °C) of methyl 2,4,6-trimethylbenzoate **A2**. Note, the quartet and singlet at 4.11 and 2.03 ppm belong to residual ethyl acetate.

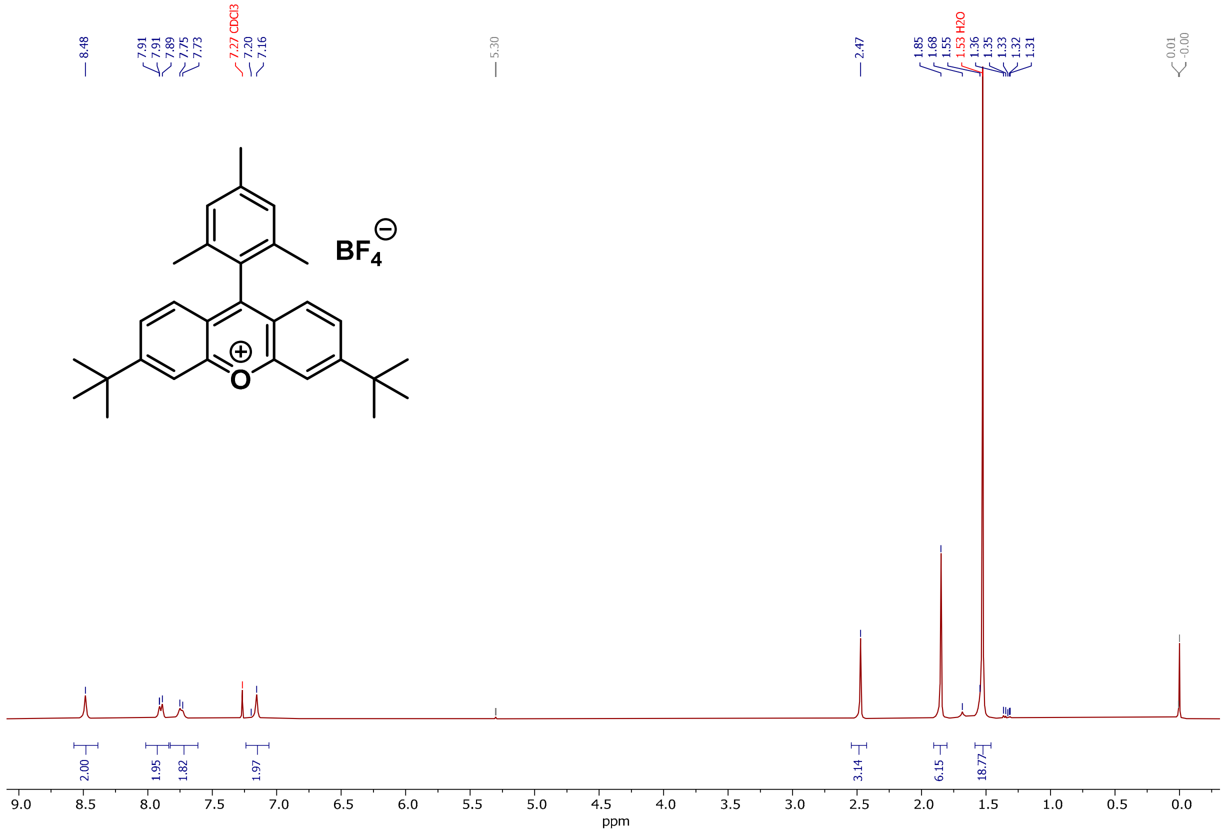

Figure S40: ^{1}H NMR spectrum (400 MHz, $CDCl_3$, 25 °C) of 3,6-di-tert-butyl-9-mesitylxanthylium tetra-fluoroborate **A3**.

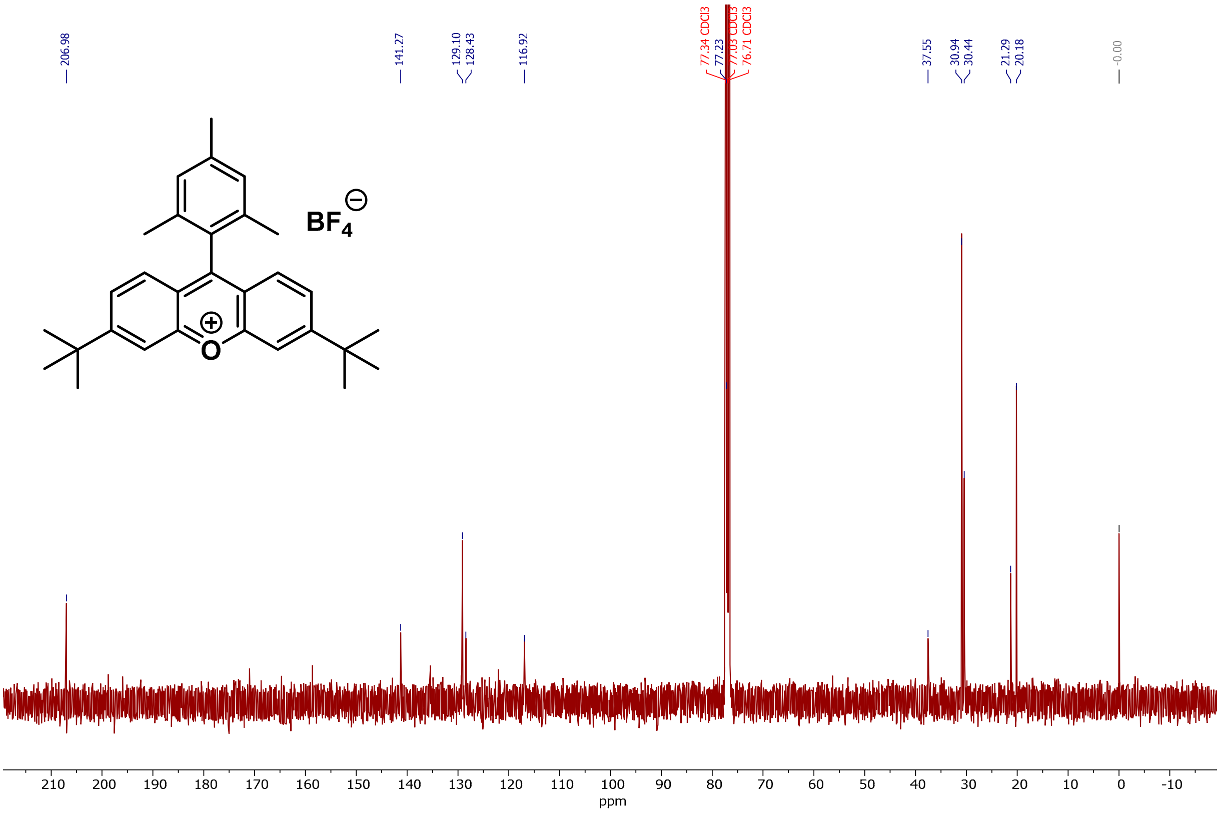

Figure S41: ^{13}C NMR spectrum (100 MHz, $CDCl_3$, 25 °C) of 3,6-di-*tert*-butyl-9-mesitylxanthylium tetrafluoroborate **A3**.

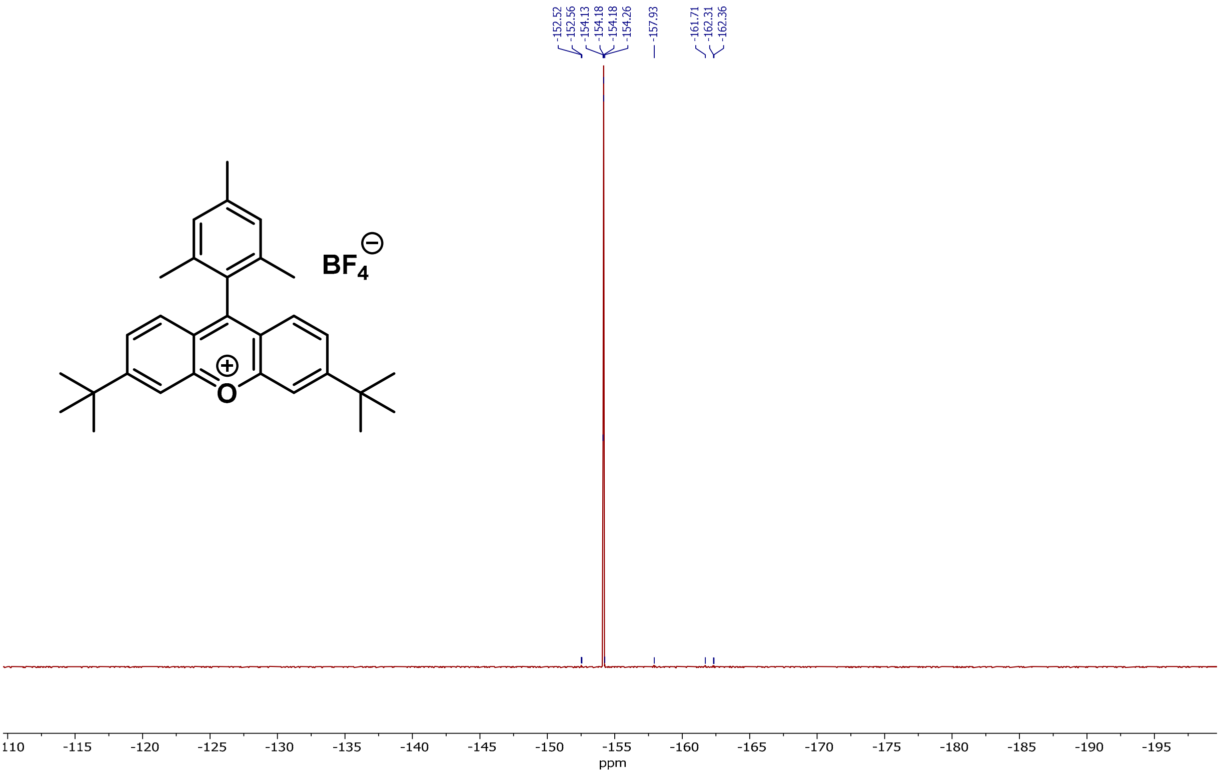

Figure S42: ^{19}F NMR spectrum (376 MHz, $CDCl_3$, 25 °C) of 3,6-di-*tert*-butyl-9-mesitylxanthylium tetrafluoroborate **A3**.

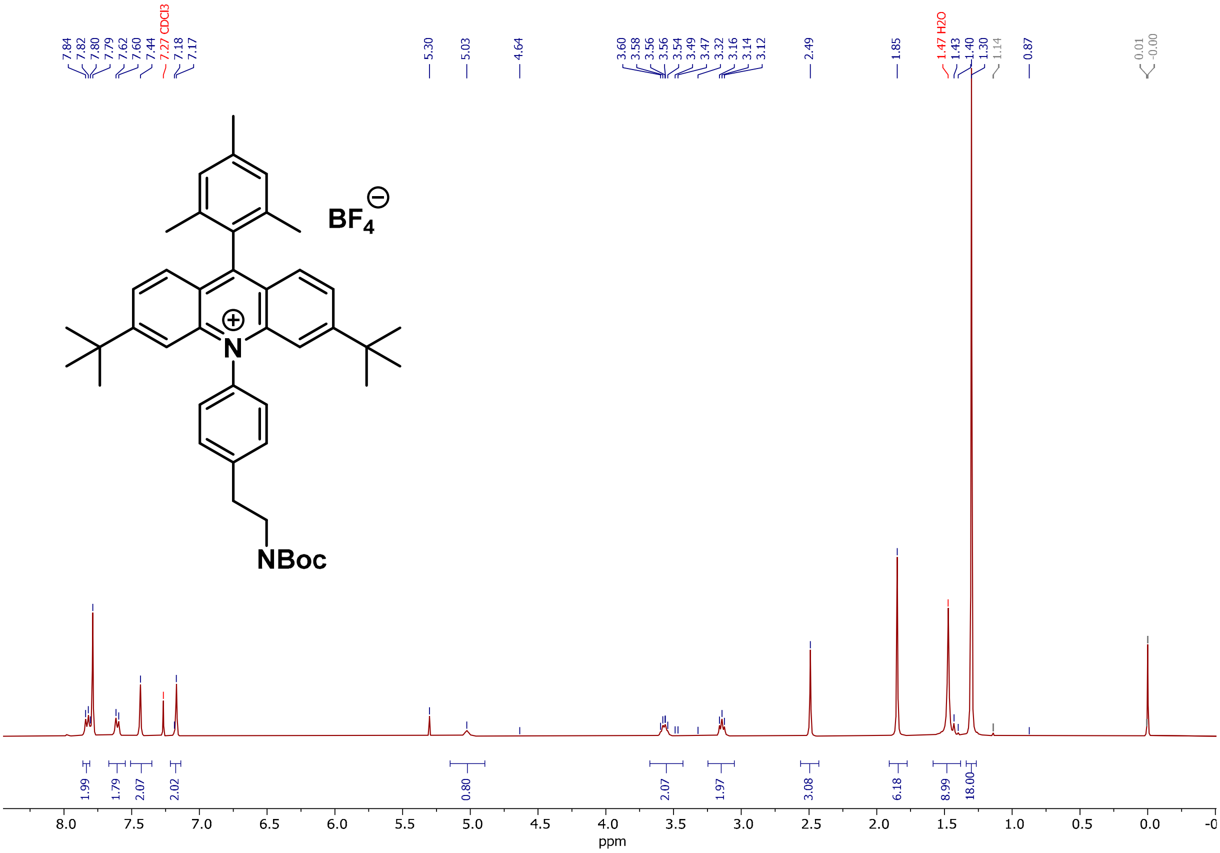

Figure S43: ^{1}H NMR spectrum (400 MHz, $CDCl_3$, 25 °C) of 10-(4-(2-((*tert*-butoxycarbonyl)amino)ethyl)phenyl)-3,6-di-*tert*-butyl-9-mesitylacridin-10-ium tetrafluoroborate **A4**.

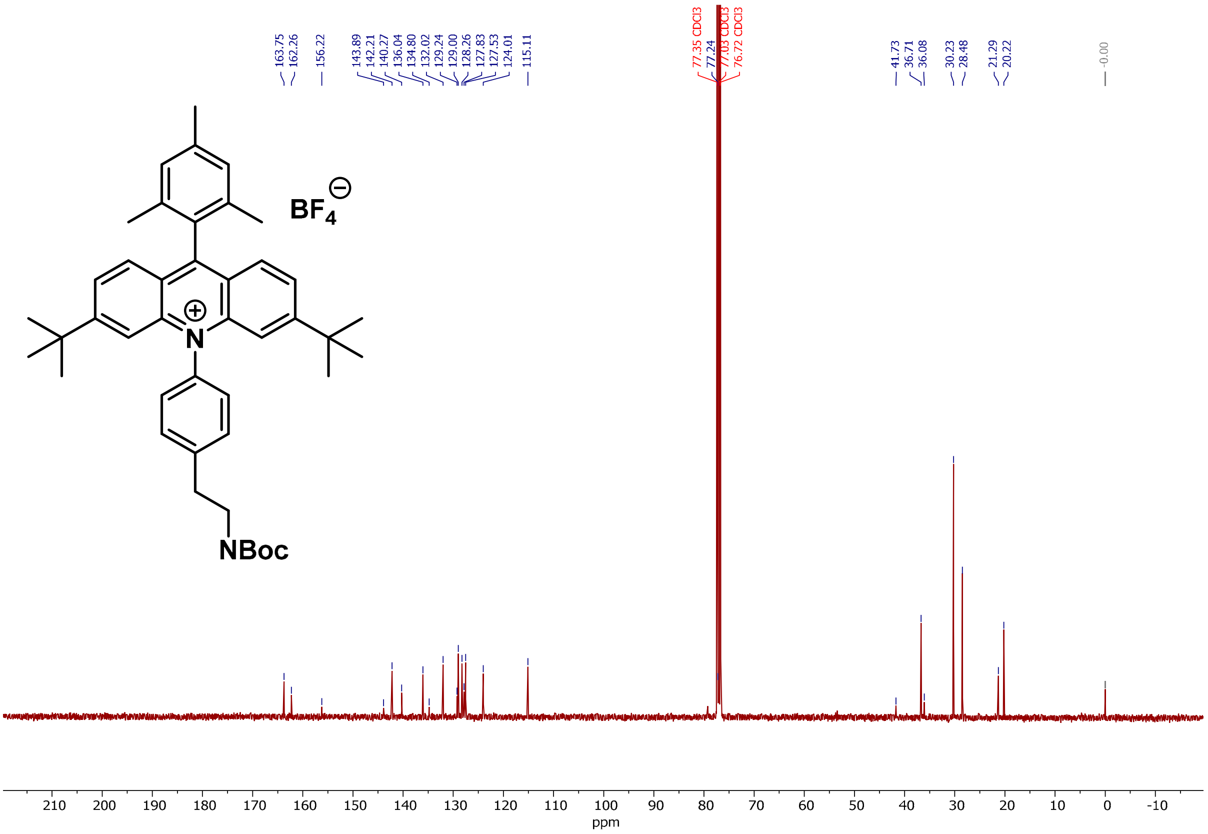

Figure S44: ^{1}H NMR spectrum (400 MHz, $CDCl_3$, 25 °C) of 10-(4-(2-((*tert*-butoxycarbonyl)-amino)ethyl)phenyl)-3,6-di-*tert*-butyl-9-mesitylacridin-10-ium tetrafluoroborate **A4**.

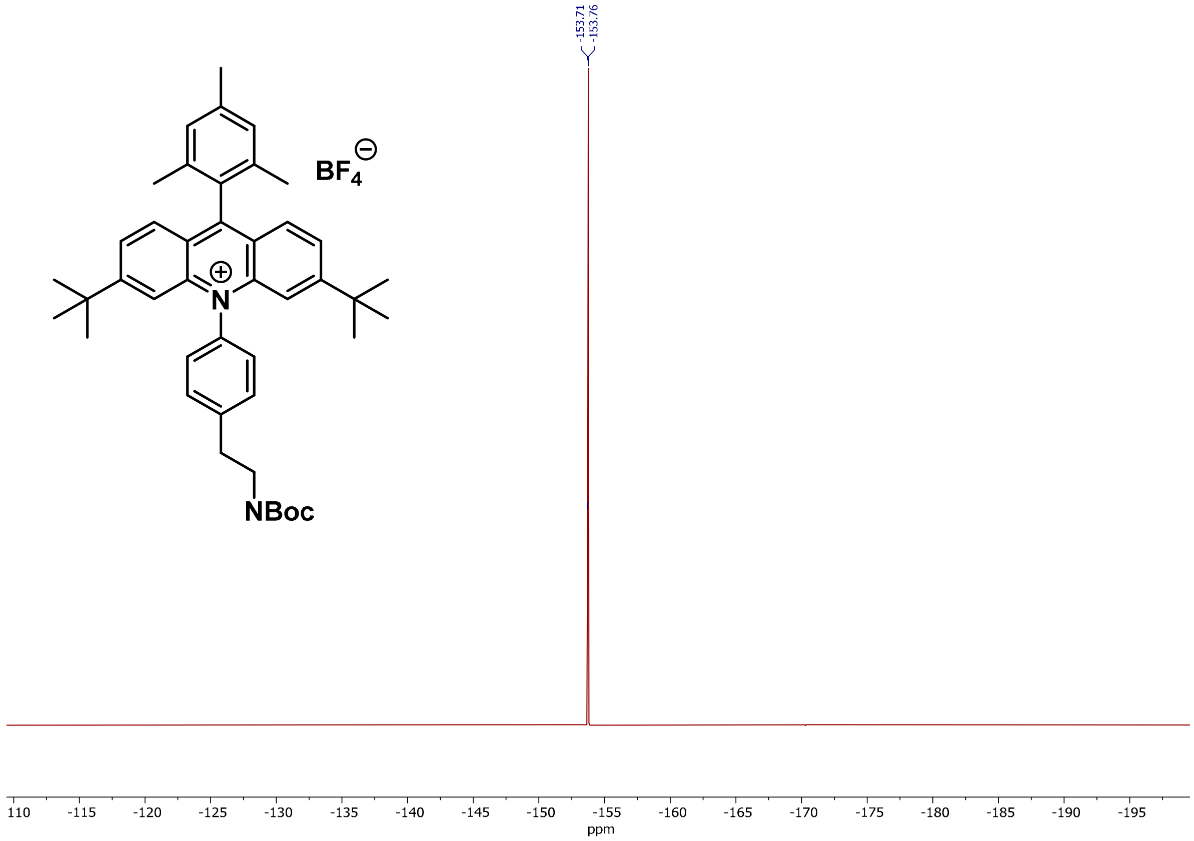

Figure S45: ^{19}F NMR spectrum (376 MHz, $CDCl_3$, 25 °C) of 10-(4-(2-((*tert*-butoxycarbonyl)-amino)ethyl)phenyl)-3,6-di-*tert*-butyl-9-mesitylacridin-10-ium tetrafluoroborate **A4**.

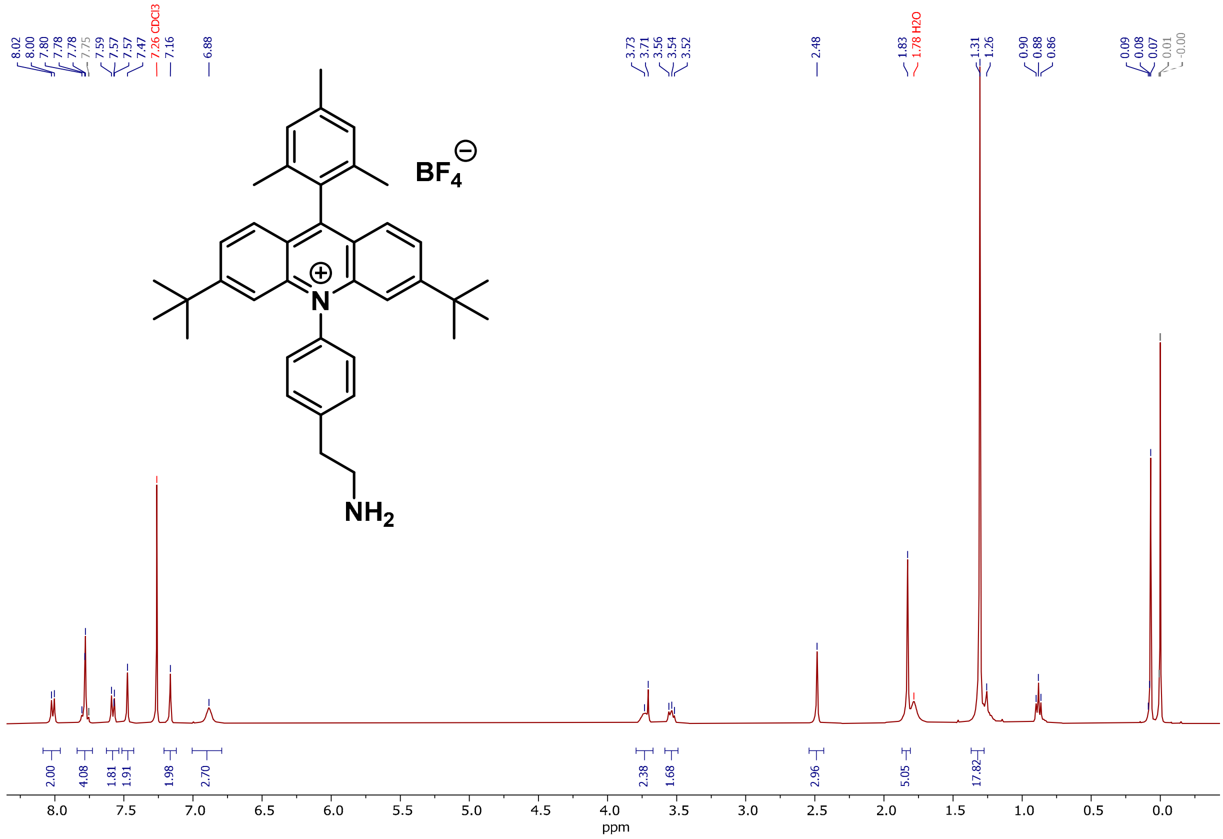

Figure S46: ^{1}H NMR spectrum (400 MHz, $CDCl_3$, 25 °C) of 10-(4-(2-aminoethyl)phenyl)-3,6-di-*tert*-butyl-9-mesitylacridin-10-ium tetrafluoroborate **A5**.

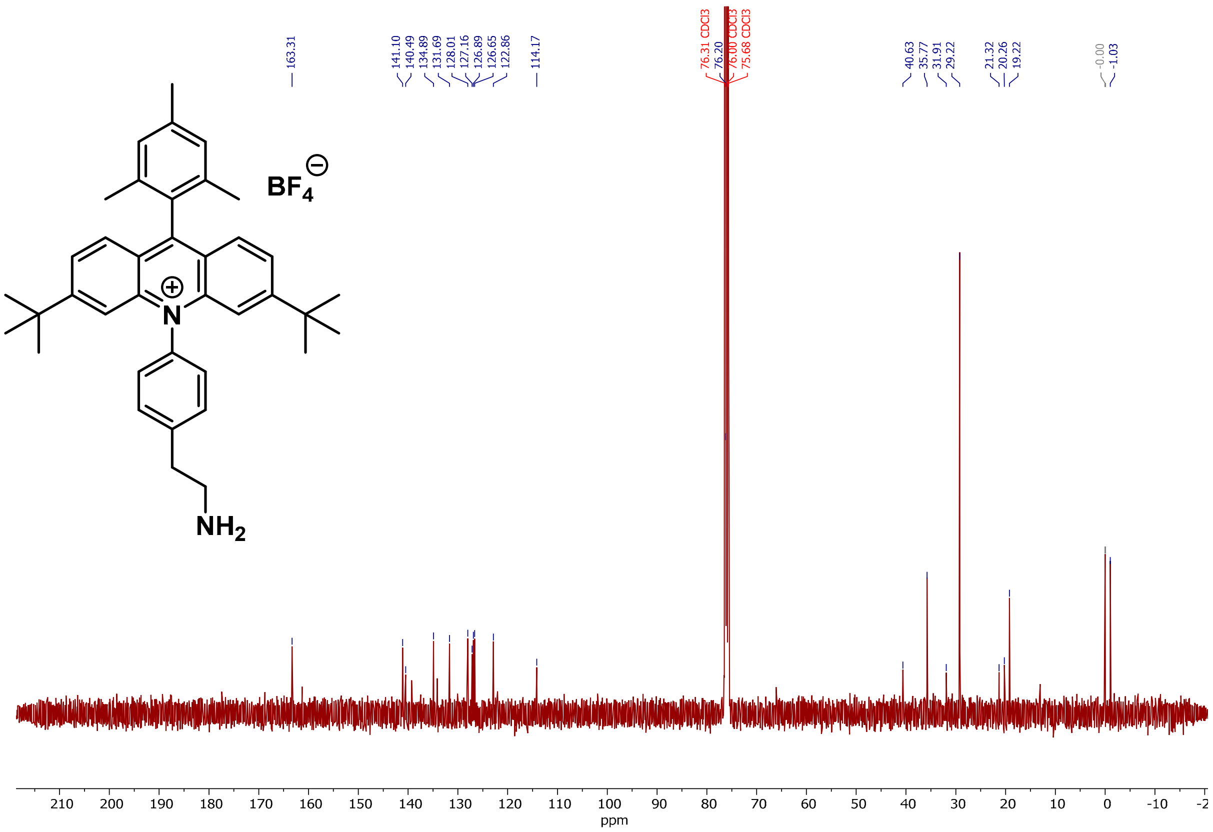

Figure S47: ^{13}C NMR spectrum (100 MHz, $CDCl_3$, 25 °C) of 10-(4-(2-aminoethyl)phenyl)-3,6-di-*tert*-butyl-9-mesitylacridin-10-ium tetrafluoroborate **A5**.

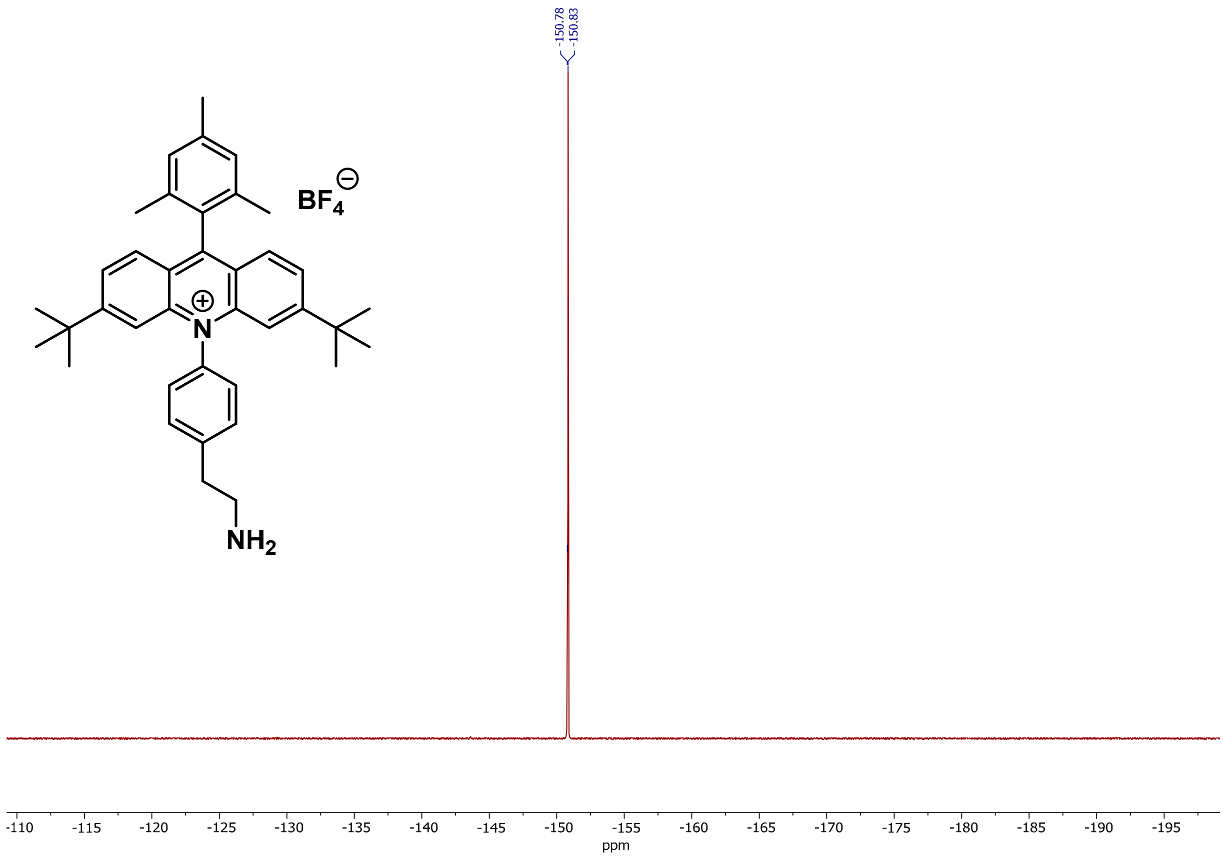

Figure S48: ^{19}F NMR spectrum (376 MHz, $CDCl_3$, 25 °C) of 10-(4-(2-aminoethyl)phenyl)-3,6-di-*tert*-butyl-9-mesitylacridin-10-ium tetrafluoroborate **A5**.
